# Weight Loss Interventions for Hispanic Women in the United States: A Systematic Review

**DOI:** 10.1155/2021/8714873

**Published:** 2021-08-19

**Authors:** Kristin E. Morrill, Melissa Lopez-Pentecost, Lupita Molina, Jeanne L. Pfander, Melanie D. Hingle, Yann C. Klimentidis, Cynthia A. Thomson, David O. Garcia

**Affiliations:** ^1^University of Arizona, University of Arizona Cancer Center, Tucson, AZ, USA; ^2^University of Arizona, College of Medicine, Department of Clinical and Translational Sciences, Tucson, AZ, USA; ^3^University of Arizona, College of Medicine, Tucson, AZ, USA; ^4^University of Arizona, University Libraries, Tucson, AZ, USA; ^5^University of Arizona, College of Agriculture and Life Sciences, Department of Nutritional Sciences, Tucson, AZ, USA; ^6^University of Arizona, Mel and Enid Zuckerman College of Public Health, Department of Epidemiology and Biostatistics, Tucson, AZ, USA; ^7^University of Arizona, Mel and Enid Zuckerman College of Public Health, Department of Health Promotion Sciences, Tucson, AZ, USA

## Abstract

**Background:**

Obesity rates in Hispanic women residing in the United States (U.S.) are disproportionately high, increasing the risk of obesity-related disease and mortality. The effectiveness of interventions targeting weight loss in this population remains largely unknown.

**Purpose:**

The purpose of this review was to systematically evaluate the evidence related to the effectiveness of weight loss interventions conducted among U.S. Hispanic women and provide guidance for future research.

**Methods:**

Bibliographic databases (*n* = 10, from each database's inception to July 2, 2019) were searched using the PRISMA guidelines for systematic reviews. Randomized controlled trials (RCTs) and quasi-experimental studies with weight change outcomes were included. Results were described in a narrative synthesis.

**Results:**

5,423 articles were assessed for eligibility based on inclusion criteria; 15 studies were included in the final review. Nine trials were RCTs and six were quasi-experimental studies; all but six were pilot studies. Most studies recruited overweight or obese women with no existing medical conditions and did not follow participants beyond the intervention. All trials were delivered in-person. Intervention strategies and content and weight change outcomes were highly variable.

**Conclusions:**

RCTs with statistically powered sample sizes are needed to robustly test the effects of weight loss interventions in this population.

## 1. Introduction

Increasing rates of obesity observed in Hispanic women living in the United States (U.S.) have prompted efforts to develop, test, and refine weight loss interventions for this rapidly growing population [1]. Almost 50% of U.S. Hispanic women are obese [2] placing them at increased risk of chronic diseases such as type 2 diabetes, hypertension, cardiovascular disease, select cancers, liver disease, and their associated mortality [3–7].

Modest weight reduction in individuals with overweight and obesity has been shown to improve a range of metabolic and cardiovascular disease outcomes including glycemic control and control of blood pressure, triglycerides, and cholesterol levels [1, 8, 9]. As such, a 3–5% weight loss goal has become a standard in weight loss interventions for both prevention and treatment of chronic diseases [10]. Despite disproportionate rates of obesity, Hispanics remain considerably underrepresented in behavioral weight loss research [11]. Barriers to participation in research include language, mistrust related to health care coverage, competing demands on time, lack of childcare, fear of unintended outcomes, and fear of deportation among immigrants [12, 13]. The generally high participant attrition rates observed in weight loss interventions provides another challenge to trials that are able to successfully recruit Hispanics; however, it remains unclear if ethnicity and demographic variables related to socioeconomic status (SES) significantly predict dropout rates [14]. These challenges have led to the use of culturally sensitive recruitment and intervention strategies to engage Hispanics in clinical research [15], yet guidance on how to effectively tailor interventions for Hispanic women, particularly in relation to effective weight loss, remains unclear.

In the U.S., Hispanic women face unique barriers and facilitators related to the adoption of weight-related behaviors. Cultural practices and beliefs related to food, family traditions and values, and religion have been identified as factors that may decrease the relevancy of standard weight loss intervention strategies for Hispanic women [16, 17]. For example, interventions that rely on study participants to precisely measure food intake may not be compatible with traditional Mexican culture where foods and recipes are prepared “to taste” [17]. Lived experiences related to immigration and the dynamic acculturation process have also been identified as important factors affecting lifestyle choices of Hispanic women. Indeed, acculturation has been linked to poorer diet quality and greater use of tobacco [18–20]. Ways in which acculturation is associated with the adoption of healthier food choices may relate to perceived accessibility to healthy, affordable foods; change in legal status; presence of an extended family member; a desire to “fit in”; and health reasons [21]. In a 2011 review, Pérez-Escamilla found an inverse association between acculturation and both diet quality and obesity, although the mechanism underpinning these nonlinear relationships remains unclear [19]. Importantly, acculturation is strongly associated with positive changes in SES and access to health care, which may act as moderators or mediators of the relationship between acculturation and lifestyle behaviors [19].

Although a number of previous reviews have summarized physical activity (PA) and diabetes (i.e., prevention and therapeutic) interventions for Hispanics in the U.S., only three have summarized weight loss interventions. A 2007 review by Lindberg and Stevens summarized the findings of three weight loss interventions recruiting Hispanics/Latinos but did not summarize information related to retention strategies, attendance, intervention delivery, setting, and other study outcomes [22]. The remaining two reviews published in 2013 and 2016 summarized evidence-based obesity treatment interventions for Latino adults, both men and women, but did not include information related to recruitment and retention, theoretical frameworks, culturally sensitive intervention strategies, and other study outcomes outside of weight loss [5, 23].

The purpose of this study was to expand on other reviews by providing an up-to-date, rigorous, and comprehensive synthesis of the literature, including examining risk of bias and quality assessment. Specifically, this review summarizes recruitment methods and effectiveness, participant characteristics, intervention strategies, process measures, and outcomes related to weight change, including clinical, behavioral, and psychosocial outcomes.

### 1.1. Objectives

The goals of the systematic review were to

1. Characterize previously tested weight loss interventions in adult, Hispanic women living in the U.S.
2. Provide evidence for the effectiveness of these interventions on weight loss outcomes
3. Identify components of successful interventions (those that have achieved clinically meaningful weight loss of ≥3% [24])
4. Identify areas for future research and provide suggestions for investigators seeking to develop weight loss interventions for Hispanic women living in the U.S.

## 2. Methods

The current systematic review was prepared according to the Preferred Reporting Items for Systematic Reviews and Meta-Analyses (PRISMA) Statement for reporting systematic reviews and meta-analyses. The protocol for this review was registered in advance with the International Prospective Register of Systematic Reviews (PROSPERO) (Registration Number: CRD42019119094). A detailed protocol for this systematic review has been published elsewhere [25].

### 2.1. Search Strategy and Eligibility Criteria

We comprehensively searched the following databases from each database's inception to July 2, 2019: PubMed, Embase, Scopus, Web of Science (Science Citation Index and Social Sciences Citation Index), PsycINFO, CINAHL, Chicano Database, SPORTDiscus, CAB Abstracts, and Google Scholar. Reference lists of included studies were searched for further references to relevant articles. We also scanned reference lists of existing reviews relevant to this systematic review for additional trials. The search was limited to publications written in the English language. See Table S1 for the full PubMed search strategy used for this review.

Inclusion criteria were defined in line with the PICOS framework and are summarized as follows:

- Population: Hispanic women, 18 years and older, residing in the U.S., studies could include participant friend(s) and/or family members
- Intervention: lifestyle interventions ≥12 weeks in duration, targeting diet and/or PA to reduce body weight
- Comparator: for randomized controlled trials (RCTs), wait-list control or usual care; for quasi-experimental studies, no comparison was required
- Outcomes: studies reporting objectively measured weight change (expressed as change in lbs or kg or body mass index (BMI) (kg/m^2^)) as a primary or secondary outcome
- Study design: RCTs and quasi-experimental studies

Studies that recruited both men and women, studies that included any number of participants who did not identify as Hispanic or Latino, studies that focused on children and/or adolescents that allowed parents to attend, and studies recruiting patients with eating disorders were excluded. Interventions that included complementary/alternative treatments or dietary supplements intended for weight loss were excluded, as well as interventions focused on preventing excessive weight gain during pregnancy. Complete exclusion criteria for each of the PICOS components outlined above is available in Table S2.

The authors would like to state that while the term “Hispanic” was used in the current manuscript to represent individuals who classify themselves as a person of Mexican, Cuban, South or Central American, Puerto Rican, or other Spanish culture or origin, regardless of race, we acknowledge the considerable heterogeneity within the Hispanic/Latino population. We included other terms (Latino/a/x, etc.) and/or subgroups (Mexican American, etc.) within our search strategy in an effort to be as inclusive as possible, and we included the terms used by each individual study. For example, if a study described participants as “Latinos,” we referred to the same participants as “Latinos” in the current manuscript.

### 2.2. Study Selection

Results from the search were uploaded by J.‐L.‐P. into Endnote citation manager software. The Endnote file was then uploaded onto the Covidence platform where it could be accessed by the authors performing the selection process. Three researchers (K.M., M.L.P., and L.M.) independently assessed the articles generated from the search strategy for eligibility. Articles were first divided into three equal sections (A, B, C), whereafter K.M. reviewed sections A + B, M.L.P. reviewed sections B + C, and L.M. reviewed A + C. The study selection process occurred in three phases: first, exclusion by title, followed by abstract, and finally by full text.

### 2.3. Data Extraction

Two authors (K.‐M. and M.‐L.‐P.) independently extracted the following data from the 15 final studies when available: author information; year of publication; study objective; eligibility information; study design; intervention characteristics; comparator and description; outcomes and time points for assessments; culturally sensitive strategies; participant characteristics (e.g., age, country of origin and acculturation); and recruitment, retention, and adherence information. Both authors independently entered this information on a standardized data extraction template, which was piloted first by K.‐M. and M.‐L.‐P. on the first three included studies. Discrepancies in data extraction were resolved by a third reviewer (D.G.). When there were multiple publications for an included study, including protocol and lessons learned papers, data were retrieved from each in order to retrieve all information relevant to the review. If during data extraction, study information was either inadequately described or missing from the publication, K.M. contacted the publication's corresponding author up to three times to request for the information.

### 2.4. Quality Assessment

To assess study quality, the Effective Public Health Practice Project Quality Assessment Tool (EPHPP) [26] was used. The EPHPP has been validated, is reliable for use in both RCTs and quasi-experimental studies [27], and has been judged to be appropriate for use in systematic reviews of effectiveness [28]. This tool evaluates study quality by assessing six different domains: (1) selection bias; (2) study design; (3) confounders; (4) blinding; (5) data collection; and (6) withdrawals/dropouts. Based on the results from these six domains, this tool then assigns studies with one of three global ratings: “weak,” “moderate,” or “strong.” Two authors (K.‐M. and D.‐G.) independently assessed the quality of each of the final 15 studies and entered data into an established standardized template created for this tool. Discrepancies in ratings were discussed to arrive at a final global rating for each study.

### 2.5. Data Synthesis

Given the substantial clinical, methodological, and statistical heterogeneity present in the included studies, a meta-analysis was not possible. The mixture of RCTs and quasi-experimental studies involving different populations (e.g., age), intervention features (e.g., focus on diet, PA, or both, duration, and strategies), and statistical methodologies (e.g., intent-to-treat analyses, missing information related to significance, standard deviation (SD), and confidence intervals) precluded our ability to pool results. In these situations, it is recommended that a qualitative synthesis be conducted [29]. Findings are presented in data summary tables and have been narratively synthesized within the text.

## 3. Results

**Figure 1:**
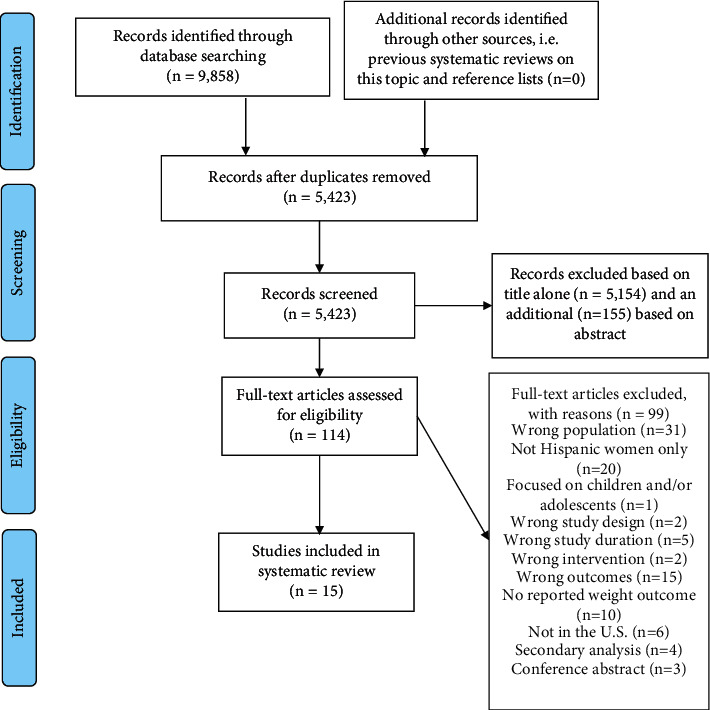
Preferred reporting items for systematic reviews and meta-analyses flow diagram.

The searches of the 10 databases retrieved 9,858 articles. Our searches of other resources (e.g., reference lists) identified no additional studies that appeared to meet the inclusion criteria. Once duplicates were removed, a total of 5,423 records remained and were screened. We excluded 5,309 records based on titles and abstracts. We obtained the full text of the remaining 114 articles. Five studies had additional manuscripts (protocol paper, lessons learned paper, etc.) that provided information relevant to the review [30–34].  Figure 1 presents a complete flow diagram, which resulted in the final 15 studies being summarized in this review [17, 35–48].

### 3.1. Characteristics of Included Studies

The characteristics of included studies are presented in Table S3. Of the 15 final studies, publication dates ranged from 1992 to 2019 with eight (53%) published in 2013 and later [35, 36, 41–44, 46, 47]. Of the included studies, nine were RCTs [35, 37–39, 41, 42, 45, 47, 48] (including two cluster RCTs) and six were quasi-experimental studies utilizing a pre-/post-test design [17, 36, 40, 43, 44, 46]. Nine (60%) of the studies were pilot studies [17, 36, 37, 39, 42–44, 46, 47]. Study sample sizes ranged from 15 to 436 participants with six (40%) having sample sizes below fifty [17, 36, 39, 42, 44, 46]. Of the 15 studies, five (33%) included participant family members or a friend [37, 38, 42, 45, 47]. One study recruited mothers with a young child [37], two recruited mothers and daughters [45, 47], one included mothers and their families [38], and another study recruited a participant plus their close friend or “comadre” [42].

The durations of the trials reviewed are as follows: four (27%) interventions were for 12 weeks [36, 42, 45, 46], one (7%) was for 16 weeks [47], one (7%) was for 20 weeks [39], three (20%) were for 6 months [37, 41, 43], three (20%) were for 12 months [17, 38, 44], two (13%) were for 24 months [35, 48], and one (7%) was for 12 weeks (first year) and then 16 weeks (second year) [40]. The majority of studies (12/15; 80%) had no follow-up assessment after the active intervention was completed; one had a 12-week follow-up [42], and two had 3-month follow-ups [37, 41].

Eligibility criteria within the included studies also varied widely. Twelve trials (80%) recruited participants with no reported existing medical conditions [17, 35–43, 45, 46], while two trials recruited participants with type 2 diabetes (13%) [47, 48], and one recruited participants with prediabetes [44]. Of the studies reviewed, six trials (40%) had no weight or BMI inclusion criteria [35, 36, 40, 45, 46, 48], six (40%) recruited individuals with BMI ≥25 kg/m^2^ [37, 39, 41, 43, 44, 47], one study recruited individuals with BMI ≥30 kg/m^2^ [17], one study recruited individuals with BMI between 27 and 50 kg/m^2^ [42], and one recruited individuals who were 20–100% above their ideal body weight [38]. Some studies were designed with minimal eligibility criteria as a means to increase the reach of participants and enhance generalizability of findings [17, 48].

### 3.2. Intervention Characteristics of Included Studies

The intervention characteristics of included studies can be found in Table S4. Twelve (80%) of the studies tested the effects of a diet plus PA intervention [17, 36–38, 41–48], while two (13%) focused only on PA [35, 40], and one (7%) focused only on diet [39]. Of the nine RCTs included in the study, comparator groups varied and included standard care control [39, 48], manual only/mailed handouts [38, 47], wait-list control [37], attention-control groups [35, 41], minimal intervention control groups [45], and the same intervention plus or minus a friend [42].

In general, details in reporting of the intervention setting, delivery, and materials varied among the 15 studies. The majority of interventions took place in community-based settings such as churches, parks, community centers, schools, and community-based organizations. The intervention setting was not reported or described in detail (e.g., “community setting”) in four (27%) of the studies [17, 36, 38, 47, 48]. Two (13%) included home visits as part of the intervention [41, 47]. All of the included trials were delivered in-person and interventionists included *promotoras* [35, 37, 39, 41, 43, 44], nursing students [36], community health educators [37, 46], registered dieticians [38, 42, 45, 48], the primary researcher [39], research assistants [39], clinicians [17], lifestyle community coaches [47], and teams of experts in psychology, counseling, nutrition, and exercise [45]. One trend observed among studies was the use of *promotoras de salud* whose roles included recruitment, leading education classes, translating during classes, conducting motivational interviewing calls, helping participants set goals, calling participants who missed classes to check in, and conducting audits of built environment in order to serve as a community advocate for environmental change. Of the six studies utilizing *promotoras*, most (*n* = 4) included in-depth descriptions of the study's *promotora* training process [30, 41, 43, 44].

All of the included studies contained group-based classes or meetings while two (13%) also included the use of motivational interviewing by phone [35] or in groups [36], and two others included the use of Individual Teaching and Coaching [41] and booster telephone calls [47] as part of the intervention. Two trials offered in-person maintenance sessions, consisting of additional monthly sessions over four [43] or six [38] months. Of the 15 trials, eight (53%) reported specific nutrition goals related to calorie reduction [38, 42, 47] or reducing or increasing consumption of certain food groups [36–38, 42, 45, 46, 48]. Eight (53%) reported specific PA goals related to minutes or steps of PA per day or week [36, 37, 41, 42, 44, 46–48]. Three (20%) studies reported a standard [43, 44] or personalized weight loss goal [47] for participants.

Of the 15 included trials, six (40%) reported a theoretical framework for the intervention [17, 35, 37, 43, 45, 46]. Of these six, one was guided by the Social-Ecological Model (SEM) and tested a multilevel intervention targeting each of the four layers of the framework (i.e., individual, interpersonal, organizational, and environmental) [35]. Five trials reported guiding their intervention with Social Cognitive Theory (SCT) [17, 37, 43, 45, 46], and one of these four combined SCT with Self-Determination Theory [43]. Three trials (20%) reported modeling their intervention after the DPP [42, 44, 47] but made no mention of a specific theoretical framework.

### 3.3. Outcomes of Included Studies

Outcome information of the included studies is summarized in Table S5. Mean age of participants across studies ranged from 27.8 to 58 years. Proxy measures of acculturation were most often utilized by studies and included participants' country of origin, preferred language (English or Spanish), generation status, and average years in the U.S. Instruments used to assess the level of acculturation included the Acculturation Rating Scale for Mexican Americans (ARMSA) [49], the Acculturation Rating Scale for Mexican Americans-II (ARSMA-II) [50], a 5-item scale (General Acculturation Index) [51], and the Short Acculturation Scale for Hispanics [52]. One study did not report any measures of acculturation [36]. The majority of studies included primarily participants of Mexican descent [17, 35, 37–39, 41, 43, 45, 47, 48], and a smaller number included participants of Dominican [42], Columbian [42], Caribbean [44], Puerto Rican [40, 42], Honduran [39], or Central American [44] descent. Two studies did not report participant country of origin [36, 46].

Within the 15 studies, considerable heterogeneity was observed in study outcomes. To be included in the review, studies needed to include weight as a primary or secondary outcome; however, other study outcomes included a wide range of clinical, behavioral, and psychosocial outcomes.

### 3.4. Clinical Outcomes

Most studies (9/15; 60%) specified change in BMI [35–37, 45, 48] or body weight (in lbs or kg) [39, 42, 44, 47] as primary or secondary outcomes; six studies included both BMI and body weight [17, 38, 40, 41, 43, 46], although one of these studies did not report the magnitude of change in BMI [40]. Aside from body weight/BMI, the most common clinical outcomes reported were waist circumference and hemoglobin A1c (HbA1c), followed by blood pressure, fasting glucose, and lipids. Importantly, one goal of this systematic review was to identify successful weight loss interventions based on a predefined measure of ≥3% reduction in initial weight. This categorization of interventions could not be performed for two reasons: (1) the large number of studies that only reported BMI, not body weight (33%), and (2) the small number of studies (27%) that reported weight loss as a percentage of baseline weight.

### 3.5. Behavioral Outcomes

Diet and PA outcomes varied widely across the 15 studies. All dietary intake outcomes were self-reported and included assessment of intake of individual food groups, total calories, % of total calories from different macronutrients, and glycemic load. The most common diet-related outcome was fruit and vegetable consumption (3/15; 20%) [17, 46, 47]. PA outcomes included self-reported measures, objective measures, and measures of physical and cardiorespiratory fitness. Of the eight studies that included PA-related outcomes [35, 36, 41–43, 45, 46, 48], half of the studies exclusively relied on self-reported measures (4/8; 50%) [36, 42, 43, 48].

### 3.6. Psychosocial Outcomes

Nine of the fifteen total studies included psychosocial outcomes with the most common being stress [36, 37, 40, 43, 44, 48]; social support [40, 42, 44, 47, 48]; and self-efficacy related to diet, PA, and weight [37, 42, 46, 48]. Five studies assessed changes in social support during the intervention and significant improvements in social support were found in three of these studies [42, 47, 48]. In addition, in one study, a positive relationship was observed between weight loss at 12 weeks and friend social support for eating habits [42]. In all nine trials, statistically significant improvements in one or more psychosocial outcomes were observed by the end of the intervention.

### 3.7. Effectiveness of Interventions on Weight Loss

#### 3.7.1. Pilot Studies

Of the nine pilot studies, five used a quasi-experimental pre-/post-test design. Among the 12-week pilot interventions, one trial reported a significant mean decrease in weight (−1.5 kg, 95% −2.5–0.5) (*p* = 0.009) [46] and the other reported a significant mean decrease in BMI (−3.22 kg/m^2^, SD not reported) (*p* ≤ 0.05) [36]. However, reported mean change in BMI reflected only the 38/48 women and 11/15 women who completed outcome assessments, respectively. Within the three remaining quasi-experimental pilot studies, only two reported significant weight loss [17, 44]. In a 12-month diet plus PA intervention with Mexican American women, one study reported a significant weight loss at 12 months of −7.2 kg (SD ± 6.8) (*p* < 0.0001) in women who completed the intervention and did not become pregnant which accounted for 55% of their sample (26/47) [17]. The authors also calculated the percentage of participants who lost ≥5% of their baseline weight (assuming no weight loss for the 14 participants who were lost to follow-up) and found this number to be 74% of the 40 enrolled participants [17]. The second 12-month pilot intervention reported a mean weight loss of 10.8 lbs (95% CI: −5.6, −16.0) (*p* < 0.001) with 58% of the participants achieving ≥5% weight loss and 42% achieving the ≥7% weight loss goal by the end of the intervention [44]. Greater than half of the completers (11/19) achieved ≥5% weight loss [44].

Among the four RCT pilot studies, only a diet plus PA intervention led to significant weight loss compared to control [47]. In one of the trials that led to differences in weight change between conditions (although changes were not statistically significant), it is worth noting that an intent-to-treat analysis was used and the control group also experienced weight loss, albeit less than half of the weight loss by the intervention group, by the end of the study [39]. In another RCT pilot study, investigators compared the effects of a 12-week behavioral weight loss intervention in a Partner Lifestyle Group (PLG) consisting of female friends/partners (or “comadres”) to the same intervention with the women alone in the Individual Lifestyle Group (ILG) [42]. Authors performed an intent-to-treat analysis to account for unavailable data [42]. At the end of 24 weeks (12 weeks post-intervention), the PLG and ILG achieved a significant weight loss of −4.7 kg (SD = 5.0) (*p* < 0.01) and −5.0 kg (SD = 6.4) (*p* < 0.01), respectively [42]. However, when compared to ILG, weight loss in the PLG was not statistically significant [42]. Notably, almost 50% of all enrolled participants achieved the weight loss goal of 5% [42]. In the only RCT pilot study that led to significant weight loss compared to control, mother and daughter dyads in the intervention arm lost significantly more weight than control dyads; however, mothers in the control group experienced a weight gain of 1.3 lbs during the 16-week study, which may have influenced the results [47]. In the last RCT pilot study, no significant difference in weight loss was reported between the intervention and wait-list control conditions; however, retention figures and sample sizes for each condition were not reported [37].

#### 3.7.2. Nonpilot Studies

In the remaining six studies, three different study designs were utilized and half of the studies did not report key details regarding weight loss outcomes including magnitude of weight or BMI loss and SD. Three of these six interventions led to significant weight loss compared to baseline [40] or control [35, 48]. In a 12- and 16-week PA intervention, participants experienced a mean weight loss of 2 lbs (SD and significance not reported) and significant reduction in BMI (*p* = 0.001); however, the magnitude of change from baseline was not reported [40]. In addition, results reflect only completers, which accounted for only 52% (118/225) of the study sample [40]. Two cluster RCTs tested the effects of a diet plus PA [45] and a PA alone intervention [35]. In the 24-month PA alone intervention, only 12-month data are reported [35]. At 12 months, although significant differences were observed in BMI between the intervention and attention-control group, the control group experienced a considerable increase in weight compared to the intervention group [35]. In addition, almost half of the intervention participants failed to attend any of the PA classes, bringing into question whether conclusions can be made about intervention effectiveness [35]. In a 12-week cluster RCT, two schools were randomized to either a diet plus PA intervention or minimal intervention control [45]. In the study, no significant changes in BMI were reported and the magnitude of BMI change was not reported [45]. In the largest RCT of the included studies (*n* = 280), women randomized to a diet plus PA intervention experienced a greater reduction of BMI than the standard care control after an intent-to-treat analysis was performed to account for missing data [48]. However, significant differences in BMI at baseline between arms were not controlled for in the analysis, which may have influenced findings [48]. In another RCT to test the effects of added family members in a diet plus PA intervention, women randomized to the Individual Group (IG) and Family Group (FG) experienced significantly greater reductions in BMI than the manual only control [38]. While the FG experienced a greater 12-month reduction in BMI than the IG, this difference was not significant [38]. In one of the few interventions to include a follow-up assessment, participants in the intervention arm experienced a weight loss of 1.46 lbs and 2.25 lbs at six and nine months (follow-up), respectively. However, no significant differences were observed between intervention and control arms [41].

### 3.8. Culturally Sensitive Strategies

The most common culturally sensitive strategies employed by the 15 included studies were the use of bilingual and bicultural research staff, intervention delivery in Spanish, translated materials, intervention content that reflected Hispanic culture (e.g., traditional and common foods, Latin dancing and Zumba, health of the family), the inclusion of family members, PA for the home, and the use of a Community Advisory Board (CAB) during intervention development and/or adaptation. Other strategies included flexible scheduling, family events before or after the intervention, having the enrollment visit in a group setting, minimal written materials, intervention materials that included topics such as traditional health and food beliefs and topics related to immigration, the inclusion of a friend or “comadre,” and tailored program planning based on acculturation (Table S4).

### 3.9. Process Evaluation and Program Evaluation

Overall, information on recruitment strategies and effectiveness were not well described. Most studies included descriptions of recruitment strategies in their methods but failed to report on each strategy's relative effectiveness. This precluded our ability to identify which specific recruitment strategies were most effective. Of the 15 studies, the most common recruitment strategies included face-to-face at churches, community centers and clinics, media (e.g., radio and T.V.), and health fairs. In one study that did report on recruitment strategy effectiveness, Lindberg et al. found that about half of the participants learned about the study from family and friends and half were recruited via flyers [17]. In another study by Seguin et al., the use of a CAB, who recruited from churches, school, and community events, yielded the most participants [46]. In another study, it was found that study staff were considerably more effective in enrolling participants through recruitment calls than a third party hired onto the study [48]. Overall, many studies did not report either one or more of the following: recruitment goals, duration of recruitment efforts, and numbers of individuals interested and screened.

Many studies evaluated retention, attendance, program fidelity, and acceptability by including process measures throughout the intervention. Ten (67%) studies sought participant feedback via mid-intervention and/or post-intervention surveys/focus groups/interviews to assess acceptability of the intervention [31, 33, 35, 40–44]. Common feedback among participants across studies was a desire for more or longer nutrition and exercise sessions [31, 44, 46] or additional content [43]. In addition, women across studies seemed to enjoy and desire more group-based activities [31, 44, 46]. Five (33%) studies reported strategies to assess intervention fidelity including completion of logs/surveys/checklists by *promotoras* [30, 43] or class leaders [46], study staff meetings [30, 41], and/or observation of intervention sessions by an additional study staff member [44]. Intervention fidelity was reported to be acceptable across these five studies. Five (33%) studies included additional analyses to identify potential moderators, mediators, and dose-response effects [35, 39, 41, 42, 44]. One study found that those who attended more classes and completed more teaching and coaching contacts experienced significant reductions in BMI, weight, and waist circumference compared to those with less intervention contacts [41]. Another study found self-weighing to be significantly associated with weight loss when study arms were combined [39]. Although not directly related to weight or BMI, one study found greater class attendance to be associated with adherence to 2008 Physical Activity Guidelines and smaller waist circumference [35].

Three studies evaluated the effect of greater intervention attendance and/or adherence on weight loss [41, 42, 44]. In one study, investigators split the intervention arm into high-intensity and low-/medium-intensity groups based upon class attendance and coaching contacts completed, and found that those in the high-intensity group experienced significantly greater decreases in BMI, weight, and waist circumference from baseline (over time) compared to the low-/medium-intensity group [41]. In two other studies, weight loss at the end of the intervention was positively associated with the number of sessions attended [42, 44] and the number of diaries submitted [42].

Overall, retention in the included studies ranged from 51% [38] to 100% [42]. One study did not report retention data [37]. The most commonly used retention strategies included study visit and class reminders, following up with participants after they missed a visit, financial incentives and raffles, flexible scheduling, providing transportation to study visits, and encouraging close contact of study staff with participants. Many studies did not report reasons for attrition; however, in the ones that did, most were related to work and time conflicts. Four (27%) studies compared characteristics of completers and noncompleters. Two studies found no significant differences between the two groups [35, 41], while the remaining two both found completers were more likely to be older [40, 48].

### 3.10. Risk of Bias and Quality Assessment

**Figure 2:**
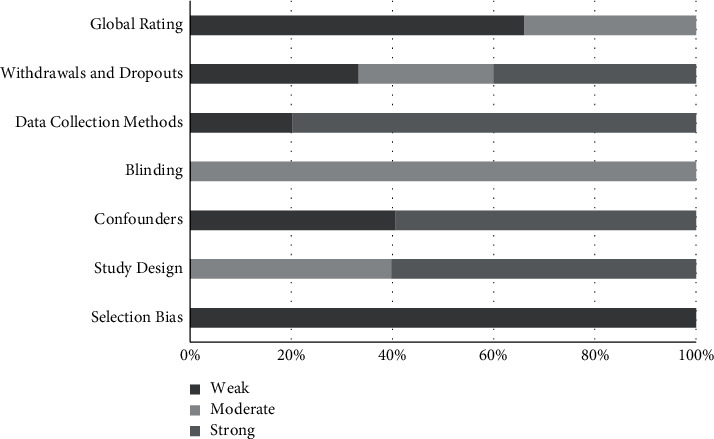
Summary of risk of bias and quality assessment. Global rating refers to the final assigned score for the study based on the five individual domains. All domains are weighed equally. Withdrawals and dropouts assess if retention or attrition figures were reported and how many participants completed the study. Data collection methods assesses if the data collection tools for the study were valid and reliable. Blinding assesses whether participants were blind to the research question and if outcome assessors were aware of the intervention or exposure status of participants. Confounders assess whether differences between groups at baseline were controlled for in the study design or analysis. Study design assesses whether the groups were randomized, how they were randomized, and if this was appropriate. Selection bias assesses if study participants are likely to be representative of the target population and the percentage of selected individuals who agreed to participate.

A summary of risk of bias and quality assessment for all included studies is shown in Figure 2 (individual study ratings are summarized in Table S6). Overall, of the 15 studies, 10 were classified as “weak” [17, 36–40, 42, 44, 46, 48] and five as “moderate” [35, 41, 43, 45, 47]. All studies received a “weak” rating for selection bias due to (1) self-selection as participants in all of the included studies given they were community members volunteering to take part in the study and/or (2) low or unreported agreement in percentage of eligible individuals agreeing to participate. Within the study design domain, all RCTs were classified as “strong” while all quasi-experimental studies were classified as “moderate.” Given a lack of control group in the quasi-experimental studies, it was expected that these studies would score lower as they provide weaker evidence when compared to RCTs. Blinding in all of the studies was scored as “moderate” due to (1) a lack of description as to whether the outcome assessor(s) was blinded to the intervention and/or treatment arm and/or (2) lack of description as to whether study participants were aware of the research question. Notably, 5 out of 6 quasi-experimental studies did not adjust for confounders and therefore received a “weak” rating in the confounders' domain. In general, studies used data collection methods for height and weight that have been validated and found to be reliable. With regard to participant withdrawals and dropouts, studies that reported ≥60% of participants completing the study and ≥80% of participants completing the study were scored as “moderate” or “strong,” respectively. Studies that reported <60% follow-up rate or those that did not report retention or attrition figures were scored as “weak.” Across studies, main areas for improvement were related to self-selection of participants, lack of adjustments for confounders, blinding of outcome assessors, high dropout rates, and lack of description regarding participant withdrawals.

## 4. Discussion

The findings from this systematic review revealed limited weight loss interventions for Hispanic women in the U.S. Among the 15 included studies, eight led to significant improvements in either BMI or weight from baseline [17, 36, 44, 46] or compared to the control arm [35, 40, 47, 48]. However, the majority of studies were short in duration and pilot in nature. Considerable heterogeneity in study design, control groups, participant characteristics, intervention format and materials, and study outcomes was found across studies. A wide variety in intervention strategies has also been observed in other systematic reviews of lifestyle and PA interventions in U.S. Hispanics/Latinos [5, 23, 53, 54]. Of the 15 included studies, 40% were published in the past five years (since 2015), displaying a potential growing interest in developing and testing weight loss interventions focused on Hispanic women. However, despite this growing trend, a paucity of lifestyle interventions targeting weight loss in Hispanic/Latino populations has been identified by previous research [11, 55]. Notably, a review by Haughton et al., evaluated the representation of racial/ethnic subgroups in behavioral weight loss interventions conducted between 2009 and 2015, and found that Hispanic/Latino populations accounted for less than 9% of participants while non-Hispanic Whites accounted for almost 60% [11]. Overall, the lack of standardized reporting of weight loss, including different weight outcomes (BMI vs. weight) and inconsistent reporting of SD, standard error, and *p* values, makes it challenging to evaluate effectiveness across studies. This heterogeneity will likely provide obstacles for future meta-analyses, which are needed to assess the totality of the evidence. Furthermore, most of the articles included in the current review were pilot studies with small sample sizes. It is important to recognize that pilot studies should focus on feasibility, process, and description rather than comparisons between groups of outcomes [56, 57]. By nature, pilot studies usually have small sample sizes and are measured more descriptively and qualitatively than RCTs [56]. In addition, small pilot studies commonly either overestimate or underestimate the true effect size of the intervention [57, 58]. Given the large number of pilot studies in the current review, it is important to acknowledge what pilot studies can and cannot provide, and given these implicit limitations, we must be careful when assessing clinical and behavioral endpoints not related to feasibility and validity. For this reason, we chose to summarize effectiveness of the interventions in pilot studies separately.

Study duration among the included articles varied widely. As expected, pilot studies tended to be shorter in duration and the vast majority of trials included in the review did not have follow-up periods after the intervention had ended. Importantly, a 2007 systematic review of weight loss-focused RCTs found that maximum weight loss tended to occur during the first 6 months and plateaued at approximately 6 months [59]. Authors of this review also concluded that participants in the clinical trials appeared to benefit from continued follow-up support that included monthly, biweekly or weekly, and quarterly face-to-face and telephone contacts [59]. This idea was reinforced by participants in Toobert et al. who, during their exit interviews, stated that as in-person sessions faded, they felt less responsibility to themselves and to the program [48]. Future weight loss interventions in Hispanic women should target at least 6 months' duration in order to maximize weight loss and should develop plans for continued support and follow-up visits in order to assess the magnitude of weight change over time. The lack of studies with follow-up measures was also noted in previous systematic reviews of obesity treatment and PA interventions in U.S. Latinos [23, 54].

Across all 15 studies, interventions resulting in significant weight loss tended to test the effects of diet plus PA interventions and report clear PA goals. The number of combined diet plus PA interventions was expected given previous research suggesting that programs based on PA alone are not as effective as combined diet plus PA interventions for weight loss [60]. Interventions were delivered by a wide range of students, community members, and professionals, and whether or not these individuals were bilingual or bicultural was often not described. This information may be particularly relevant given previous findings from focus groups where Mexican American immigrant women expressed a strong preference for female Mexican interventionists [16, 17]. From the studies that reported adapting their interventions from the DPP or other existing programs, some did not include any further information on which specific constructs were carried over or emphasized and how they informed the study's intervention strategies. This lack of description limits the replicability of the intervention and our ability to understand causal mechanisms of change.

Social support has been identified as an important factor in the adoption of healthy lifestyle behaviors in Hispanic populations [54, 61]. A previous 2012 review by Ickes and Sharma found that increasing social support was a focus in 65% of PA interventions in Hispanic adults [54]. In the current review, participant feedback across studies stated they enjoyed group sessions [31, 44, 46] and felt that a widened social support network was a facilitator to engaging in exercise [40]. The use of *promotoras* was also found to contribute a sense of social support for participants throughout an intervention by facilitating behavior change by motivating participants and providing a sense of emotional and social support [41]. Future studies should continue to target social support as a means to facilitate the adoption of healthy lifestyle behaviors in U.S. Hispanic women, as efforts to improve social support seem to be appreciated by participants. However, the extent to which social support facilitates weight loss in diet and PA interventions remains unclear.

When conducting narrative syntheses, it is recommended that authors explore potential moderator variables to help identify how, why, and for whom interventions are working [62]. This task was particularly challenging due to the large number of pilot studies and little overlap existing between the interventions across components such as intervention duration, delivery, and strategies. Of the six studies that utilized *promotoras* to deliver the interventions, only two led to significant weight loss [35, 44]. Of these, one was a RCT (*n* = 436) testing the effects of a PA intervention which resulted in significant weight loss at 12 months compared to the attention-control group [35] and the other was a pre-/post-test diet plus PA feasibility study (*n* = 20) that resulted in significant weight loss at 12 months [44]. In a 2013 systematic review, Perez et al. found that obesity treatment interventions that yielded the largest effect sizes were delivered in a wide range of settings (e.g., Church, healthcare, community center) by differing interventionists (e.g., healthcare professionals, *promotoras*, registered dieticians) [23]. In the present review, no clear or consistent effect of *promotoras* on weight loss was observed across studies. The addition of one or more family members or friends was a common characteristic of interventions for U.S. Hispanic women. While five studies (33%) included either a family member or friend, only two (13%) tested the effects of this added member compared to the woman alone [38, 42]. In these two interventions, no additional benefit on participant weight loss was observed from the inclusion of family [38] or a close friend [42]. Overall, more research is needed to determine whether the inclusion of family or friends in an intervention promotes greater weight loss in U.S. Hispanic women.

Overall, little overlap was found in the various acculturation measures utilized and only a few used validated measures. Notably, a 2010 study by Wallace et al. identified 26 acculturation measures focused on Hispanics used in the literature [63]. Of these, only a few were found to be reliable and valid, including the ARMSA, ARSMA-II, and the 12-item Bidimensional Acculturation Scale for Hispanics [63]. Importantly, these multidimensional measures assess several aspects of Hispanic culture including but not limited to language, country of origin, and cultural identity, making them more comprehensive and favorable than using any of these measures alone [63]. Nearly all participants in the included studies were reported to be less acculturated limiting our ability to determine whether or not acculturation promoted weight loss. Our findings parallel those of a previous review of weight loss interventions in Hispanic adults where similar issues with the lack of use of standardized measures of acculturation were reported [22]. Future studies should strive to incorporate validated and reliable measures so that meaningful comparisons can be made across study populations.

The present review found that the majority of studies provided in-depth descriptions of culturally sensitive strategies utilized. This finding differed from a finding from a previous 2007 review of weight loss interventions in U.S. Hispanic populations by Lindberg and Stevens, which found that studies commonly failed to describe the culturally sensitive strategies implemented by the studies [22]. While descriptions of these strategies seem to have improved, authors should refrain from using vague terms in their description of the adaptations to enhance replicability of the intervention. In a 2013 review of obesity treatment interventions in U.S. Hispanic Latinos, Perez et al. highlighted the importance of utilizing culturally relevant strategies in obesity-related research and, when possible, developing interventions that target multiple levels of the SEM in acknowledgment of the various barriers faced by many attempting to change their diet and PA behaviors [23].

Previous weight loss intervention research has established that greater attendance and intervention completion are associated with greater weight loss [14]. Importantly, monitoring participants' intervention engagement may offer insight into why some interventions work while others do not. For example, in a dietary intervention study by Lippke et al., changes in action planning and coping planning emerged as effective mediators only if engagement in the intervention was at a certain level [64]. In the current review, despite the number of studies having participants keep food and PA logs, only one study reported the number of submitted logs as a measure of intervention adherence [42]. This makes it difficult to identify whether participants were engaging in self-monitoring behaviors and ultimately the ability of the investigators to assess whether these behaviors mediated the observed intervention effects.

A number of gaps remain regarding which intervention strategies are most effective for Hispanic women in the U.S. Based on the findings of this review, the following guidance can be provided:

1. A paucity of rigorous RCTs testing the effect of diet and PA interventions on weight loss among Hispanic women exists. This review identified several pilot studies that resulted in significant weight loss. There is a need to advance research by testing large, adequately powered interventions of ≥6 months in order to test the effectiveness of interventions on weight loss. Large RCTs should strive to address common quality issues related to self-selection of participants, blinding of outcome assessors, and high attrition rates.
2. Country of origin of participants should more routinely be collected and reported given the vast heterogeneity within the Hispanic and/or Latino population. In addition, reliable and valid measures of acculturation should be used to allow meaningful comparisons to be made across studies.
3. Reporting on recruitment, retention, attendance, and adherence should consider including information regarding which recruitment strategies were most effective and which retention strategies were most accepted and beneficial for participants.
4. Future RCTs should employ best practices for missing data to avoid biased and/or invalid scientific conclusions [65].
5. Studies should report direction and magnitude of change in weight loss and standard deviation data to allow for meta-analyses.
6. Reporting on weight loss should include crude weight and percentage of baseline body weight lost. This is because percentage weight change takes into account baseline differences in weight and height while crude weight loss does not. In addition, authors should report the number of participants who achieved 3%, 5%, and ≥10% weight loss given these markers have each been associated with clinically meaningful improvements in a range of biomarkers.
7. When appropriate, changes in diet, PA, and behavioral outcomes should be documented to allow for the analysis of potential mediators of intervention effectiveness.
8. Future research in adult Hispanic women should test approaches that overcome commonly reported barriers to participation in weight loss studies (e.g., transportation and time conflicts).
9. For replicability and transparency purposes, authors should consider publishing protocol papers to ensure important details regarding intervention delivery and strategy are fully described.

Limitations of this review include the low number of studies that included weight loss as a percentage of baseline weight. This impeded our ability to use a predetermined measure of intervention success on weight loss, which in turn led to our inability to identify characteristics of successful interventions. Similar challenges have been reported in other systematic reviews evaluating the effectiveness of weight loss interventions in Hispanic and other populations [5, 66].

Given our inclusion criteria stated that studies must have reported weight change as a primary or secondary outcome, it is likely that weight loss was not always the primary focus of an intervention included in this review. This might have been the case for some of the interventions that only targeted PA and may have implications for the design, including statistical power, and content of the interventions.

We acknowledge that limiting this review to studies that included 100% Hispanic women fails to recognize the many studies that recruited both Hispanic men and women. However, our choice to focus on Hispanic women was informed by their unique attitudes, barriers, and facilitators related to diet, PA, and weight loss that interact with unique sociocultural contextual factors including gender role strains and the immigration experience. In addition, research has shown that differences exist in key predictors of weight loss when using racial-/ethnic- and sex-specific models [67]. We feel that these factors highlight the need to focus specifically on Hispanic women.

The large number of pilot studies included in this review complicates our ability to make conclusions about the state of weight loss interventions in U.S. Hispanic women. Specifically, the small sample sizes, short duration, pre-post design, and focus, for many of the studies, on feasibility, limit our ability to conclude whether or not interventions demonstrate initial efficacy or future effectiveness. We have attempted to mitigate this issue by separately evaluating the pilot studies; however, evaluation of a greater number of RCTs is needed before any sound conclusions regarding effectiveness can be made.

Despite these limitations, a number of strengths in the review should be acknowledged. To our knowledge, this review is the first to rigorously evaluate and summarize weight loss interventions in the U.S. for Hispanic women. This included developing a comprehensive search strategy with a detailed data extraction process reflected in the review's tables. This review was also the first to use a validated and reliable risk of bias and quality assessment of included studies and summarize important process measures, which the authors determined was important given the large number of pilot studies. The large number of pilot studies reviewed could hint that a number of RCTs are currently being developed or are underway making the comprehensive list of suggestions for future research particularly timely.

## 5. Conclusions

In conclusion, weight loss interventions in U.S. Hispanic women display considerable heterogeneity in methods and reporting and are pilot in nature impeding the ability to make meaningful conclusions about their overall effectiveness. However, there seems to be growing interest in developing these interventions. This effort to summarize the existing state of weight loss interventions for this underrepresented group are particularly timely as recently the National Institute of Health's Minority Health and Health Disparities Strategic Plan for 2021–2025 was released with Strategy 1.2 being to develop and assess interventions to improve the health status of minority populations [68]. This review serves as a step towards assessing and building upon the current landscape of existing weight loss interventions for this population.

## 6. Deviation from Protocol

In efforts to increase transparency and reduce bias when summarizing the effectiveness of weight loss interventions, we set a weight loss of ≥3% baseline body weight as a marker of a successful intervention. During the data extraction process, it became clear that our predetermined measure of weight loss success could not be utilized as many of the included studies did not report percent weight change. In addition, given the current environment (COVID-19 pandemic), it was particularly challenging to connect with authors of the included studies. For this reason, we were not able to obtain the intervention materials of the included studies as stated in the protocol paper.

## Data Availability

The data supporting this systematic review are from previously reported studies and datasets, which have been cited. These data are available in the Supplementary Files.

## Abbreviations

U.S.: United States
SES: Socioeconomic status
PA: Physical activity
PRISMA: Preferred reporting items for systematic reviews and meta-analyses
RCTs: Randomized control trials
BMI: Body mass index
EPHPP: Effective public health practice project quality assessment tool
SD: Standard deviation
SEM: Social-ecological model
SCT: Social cognitive theory
DPP: Diabetes prevention program
ARSMA-II: Acculturation rating scale for Mexican Americans II
HbA1c: Hemoglobin A1c
PLG: Partner lifestyle group
ILG: Individual lifestyle group
IG: Individual group
FG: Family group
CAB: Community advisory board.

## Supplementary Materials

Supplementary Materials Table S1: PubMed search strategy. This table contains the current review's full search strategy for the PubMed database. This information is meant to increase transparency and replicability of the search strategy. Table S2: PICOS framework. This table contains the specific inclusion and exclusion criteria as part of the review's search strategy. This information is meant to increase transparency and replicability of the search strategy. Table S3: characteristics of included studies. This table summarizes the sample size, study objectives, study design, duration of intervention and follow-up assessment point(s) (if applicable), and study eligibility criteria of the 15 included studies. Table S4: intervention characteristics of included studies. This table summarizes the intervention focus (e.g., physical activity plus diet intervention, physical activity only intervention, or diet only intervention), comparator arm, intervention setting and delivery modality, intervention format and strategies, interventions goals/recommendations, culturally sensitive intervention strategies, and theoretical framework for the 15 included studies. Table S5: outcomes of included studies. This table summarizes the study participant characteristics (e.g., age, Hispanic/Latino subgroup, and acculturation), study outcomes; data collection time points; weight change (kg or BMI); additional significant findings; retention and adherence strategies; and retention, adherence, and attendance information of the 15 included studies. Table S6: risk of bias and quality assessment of included studies. This table summarizes the individual study ratings for each of the six domains found within the risk of bias and quality assessment tool utilized in the study (i.e., the Effective Public Health Practice Project Quality Assessment Tool (EPHPP)). For each domain, studies can be scored as “weak,” “moderate,” or “strong.” Scores of the individual domains can then be coalesced into an overall quality score for the study (i.e., “weak,” “moderate,” or “strong”).
