# Supplementary material for: Weight Loss Interventions for Hispanic Women in the United States: A Systematic Review
Source: J Environ Public Health. 2021 Aug 19;2021:8714873. doi: 10.1155/2021/8714873 (PMC8397533; doi:10.1155/2021/8714873)
Supplement: Supplementary Materials — Table S1: PubMed search strategy. This table contains the current review's full search strategy for the PubMed database. This information is meant to increase transparency and replicability of the search strategy. Table S2: PICOS framework. This table contains the specific inclusion and exclusion criteria as part of the review's search strategy. This information is meant to increase transparency and replicability of the search strategy. Table S3: characteristics of included studies. This table summarizes the sample size, study objectives, study design, duration of intervention and follow-up assessment point(s) (if applicable), and study eligibility criteria of the 15 included studies. Table S4: intervention characteristics of included studies. This table summarizes the intervention focus (e.g., physical activity plus diet intervention, physical activity only intervention, or diet only intervention), comparator arm, intervention setting and delivery modality, intervention format and strategies, interventions goals/recommendations, culturally sensitive intervention strategies, and theoretical framework for the 15 included studies. Table S5: outcomes of included studies. This table summarizes the study participant characteristics (e.g., age, Hispanic/Latino subgroup, and acculturation), study outcomes; data collection time points; weight change (kg or BMI); additional significant findings; retention and adherence strategies; and retention, adherence, and attendance information of the 15 included studies. Table S6: risk of bias and quality assessment of included studies. This table summarizes the individual study ratings for each of the six domains found within the risk of bias and quality assessment tool utilized in the study (i.e., the Effective Public Health Practice Project Quality Assessment Tool (EPHPP)). For each domain, studies can be scored as “weak,” “moderate,” or “strong.” Scores of the individual domains can then be coalesced into an overall quality score f [file 8714873.f1.docx]

**Title:** Weight Loss Interventions for Hispanic Women in the United States: A Systematic Review

**Authors:**

Kristin E. Morrill, University of Arizona Cancer Center, University of Arizona, Tucson, AZ, United States, Email: [morrill1@email.arizona.edu](mailto:morrill1@email.arizona.edu)

Melissa Lopez-Pentecost, Department of Clinical and Translational Sciences, College of Medicine, University of Arizona, Tucson, AZ, United States, Ph: (520) 808-4249, Email: melissalopez7@email.arizona.edu

Lupita Molina, College of Medicine, University of Arizona, Tucson, AZ, United States, Ph: (520) 234-3671, Email: molinal@email.arizona.edu

Jeanne L. Pfander, University Libraries, University of Arizona, Tucson, AZ, United States, Ph: (520) 621-6375, Email: jpfander@arizona.edu

Melanie D. Hingle, Department of Nutritional Sciences, College of Agriculture & Life Sciences, University of Arizona, Tucson, AZ, United States, Ph: (520) 621-3087, Email: hinglem@email.arizona.edu

Yann C. Klimentidis, Department of Epidemiology and Biostatistics, Mel and Enid Zuckerman College of Public Health, University of Arizona, Tucson, AZ, United States, Ph: (520) 621-0147, Email: yann@arizona.edu

Cynthia A. Thomson, Health Promotion Sciences Department, Mel and Enid Zuckerman College of Public Health, University of Arizona, Tucson, AZ, United States, Ph: (520) 626-1565, Email: cthomson@arizona.edu

David O. Garcia, Department of Health Promotion Sciences, Mel and Enid Zuckerman College of Public Health, University of Arizona, Tucson, AZ, United States, Ph: (520) 626-4641, Email: davidogarcia@arizona.edu

**Table S1: PubMed Search Strategy (07/02/2019)***

| Hispanic: | "Hispanic Americans"[Mesh] OR Hispanic[tiab] OR Hispanics[tiab] OR Central American[tiab] OR Central Americans[tiab] OR Chicano[tiab] OR Chicanos[tiab] OR Chicana[tiab] OR Chicanas[tiab] OR Cuban[tiab] OR Cubans[tiab] OR Dominican[tiab] OR Dominicans[tiab] OR Latin American[tiab] OR Latin Americans[tiab] OR Latin[tiab] OR Latina[tiab] OR Latinas[tiab] OR Latino[tiab] OR Latinos[tiab] OR LatinX[tiab] OR Mexican[tiab] OR Mexicans[tiab] OR Puerto Rican[tiab] OR Puerto Ricans[tiab] OR South American[tiab] OR South Americans[tiab] OR Spanish speaker[tiab] OR Spanish speakers[tiab] OR Spanish speaking[tiab] |
| --- | --- |
| Women: | "Women"[Mesh] OR "Female"[Mesh]) OR "Mothers"[Mesh] OR woman[tiab] OR women[tiab] OR female[tiab] OR females[tiab] OR feminine[tiab] OR mother[tiab] OR mothers[tiab] OR daughter[tiab] OR daughters[tiab] OR grandmother[tiab] OR grandmothers[tiab] OR aunt[tiab] OR aunts[tiab] OR sister[tiab] OR sisters[tiab] OR mujer[tiab] OR mujeres[tiab]) OR madre[tiab] OR madres[tiab] OR abuela[tiab] OR abuelas[tiab] OR tia[tiab] OR tias[tiab] OR hermana[tiab] OR hermanas [tiab] |
| Weight: | "Body Weight Changes"[Mesh] OR "Body Mass Index"[Mesh] OR "Body Weight"[Mesh:NoExp] OR "Body Weight Maintenance"[Mesh] OR "Ideal Body Weight"[Mesh] OR "Overweight"[Mesh] OR "Overnutrition"[Mesh] OR "Waist Circumference"[Mesh] OR "Waist-Height Ratio"[Mesh] OR body mass index[tiab] OR BMI[tiab] OR body weight[tiab] OR overweight[tiab] OR over nutrition[tiab] OR overnutrition[tiab] OR weight gain[tiab] OR weight loss[tiab] OR weight maintenance[tiab] OR weight management[tiab] OR obesity[tiab] OR obese[tiab] OR waist circumference[tiab] OR waist-height ratio[tiab] OR waist-to-height ratio[tiab] |
| Interventions: | "Diet, Reducing"[Mesh] OR "Weight Reduction Programs"[Mesh] OR "Diet"[Mesh] OR reducing diet[tiab] OR reducing diets[tiab] OR diet[tiab] OR diets[tiab] OR dietary[tiab] OR weight reduction[tiab] OR weight loss[tiab] OR losing weight[tiab] OR eating habit[tiab] OR eating habits[tiab] OR food intake[tiab] OR food restriction*[tiab] OR eating behavior[tiab] OR eating behaviors[tiab] OR “Physical Exertion"[Mesh] OR "Physical Fitness"[Mesh] OR "Exercise Therapy"[Mesh] OR "Exercise"[Mesh] OR "Sports"[Mesh] OR "Physical Education and Training"[Mesh] OR (movement*[tiab] OR physical exertion[tiab] OR physical activity[tiab] OR physical activities[tiab] OR physical fitness[tiab] OR sport[tiab] OR sports[tiab] OR exercis*[tiab] OR "Women's Health Services"[Mesh] OR "Health Promotion"[Mesh] OR "Life Style"[Mesh] OR intervention*[tiab] OR health promotion*[ tiab] OR life style*[tiab] OR lifestyle*[tiab] |
| Age: | NOT ("Child"[Mesh] OR "Infant"[Mesh] OR "Adolescent"[Mesh]) NOT "Adult"[Mesh] |

**FINAL SEARCH (combining above listed search sets):**

HISPANIC AND WOMEN AND WEIGHT AND INTERVENTIONS AND AGE

**Limit: English language only**

*This information was originally published in the protocol paper for the current review in *Systematic Reviews* by Morrill KE et al. No changes were made except to replace “Title/Abstract” to “tiab” within the search strategy. The information was also formatted into a table. This table is distributed under the terms of the Creative Commons Attribution 4.0 International License: https://creativecommons.org/licenses/by/4.0/

**Table S2: PICOS Framework***

| PICOS Strategy | Inclusion criteria | Exclusion criteria |
| --- | --- | --- |
| P – Population | Hispanic women, 18+ years old, living in the U.S. | Studies that recruited both men and women (however, studies that included men and children as a strategy to engage women will be kept), studies focused on children and/or adolescents that allowed parents to attend, patients who are hospitalized or institutionalized), patients with eating disorders, patients who have recently undergone bariatric surgery |
| I - Intervention | Lifestyle interventions ≥ 12 weeks in duration, targeting diet and/or physical activity to reduce body weight | Surgical procedures, nonsurgical devices and procedures, pharmacological treatments, complementary/alternative treatments, dietary supplements intended for weight loss, population-focused health promotion campaigns, and interventions that do not focus on modifying weight, interventions to prevent excessive weight gain during pregnancy |
| C - Comparison | For RCTs, wait-list control or usual care  For quasi-experimental, no comparison required |  |
| O - Outcome | Studies reporting objectively measured weight change (expressed as change in lbs or kg or BMI (kg/m^2^)) as a primary or secondary outcome | Self-reported measures of weight change |
| S – Study design | RCTs and quasi-experimental studies | Reviews, observational studies (cross sectional, case-control and cohort studies), case reports, case series, in vitro studies, animal studies, secondary analyses of trials, and survey development studies |

*This table was originally published in the protocol paper for the current review in *Systematic*

*Reviews* by Morrill KE et al. No changes were made to the table. This figure is distributed under the terms of the Creative Commons Attribution 4.0 International License: https://creativecommons.org/licenses/by/4.0/

**Table S3: Characteristics of Included Studies**

| **Study** | **Sample Size** | **Study Objective** | **Study Design** | **Duration of intervention/follow-up** | **Study Eligibility** |
| --- | --- | --- | --- | --- | --- |
| **Arredondo 2017**  **Arredondo 2015**  **(*Fe en Acciόn, Faith in Action)*** | n=436 | To evaluate the impact of a faith-based intervention to promote PA in Latinas | Cluster RCT | 24-month intervention/ No follow-up; 12-month data reported | Self-identify as a Latina, age 18-65 yrs. old, inactive, attend church at least 4x/month for any reason, live within 15 min. driving distance of the church, access to reliable transportation to get to the church, identify no barriers to attend activities at the church for next 24 months, not attend other churches enrolled in the study, and not have a condition that would preclude them from being active    PARmedX (if any positive responses to PAR-Q)  Women were excluded if they did not return the PARmedX |
| **Baldwin 2015**  ***(Women’s Path to Prevention (WPP))*** | n=48 | To measure the short-term effects of a healthy lifestyle, multicomponent intervention on a range of clinical and behavioral outcomes | Quasi-experimental (pretest-posttest) pilot study | Four 12-week cohorts over 2 years/No follow-up | Women 35 yrs. and older, not pregnant, able to speak and understand English or Spanish, and received health care provider approval to participate in a PA program  No exclusion criteria reported |
| **Berry 2011** | n=56 | To test the effects of a nutrition and exercise education, coping skills training, and PA intervention on clinical and behavioral outcomes in Spanish-speaking Women | RCT pilot study | 6-month intervention/3-month follow-up | BMI ≥ 25 kg/m^2^ and child between ages of 2-4 yrs.  No exclusion criteria reported |
| **Cousins 1992** | n=168 | To assess the effectiveness of a culturally-sensitive, family-based weight loss program for CVD risk reduction in obese MA women | RCT | 12-month intervention/No follow-up | Self-identify as a MA woman, age 18-45 yrs. old, and 20-100% above ideal body weight  Women were excluded if they were hypertensive, had diabetes, or had any other chronic illness with dietary or exercise recommendations that differed from the study |
| **Faucher 2010** | n=24 | The primary aim of this pilot study was to evaluate a portion control intervention compared to standard care counseling on weight loss in low-income MA women | RCT pilot study | 20-week intervention/No follow-up | BMI ≥ 25 kg/m^2^ and wanted to lose weight  Women were excluded if they had a current diagnosis with depression or anxiety disorder, were pregnant or planning to be, using steroids (including oral contraceptives), taking psychotropic, antidepressant, or beta-blocker medications, or had any contraindications for exercise |
| **Harralson 2007**  ***(Un Corazon Saludable: A Healthy Heart)*** | n=225 | To address barriers and issues surrounding PA by implementing a culturally-sensitive exercise/educational program focused on decreasing risk factors associated with CVD and metabolic syndrome | Quasi-experimental (pretest-posttest) | 1^st^ year = 12 weeks /No follow-up  2^nd^ year = 16 weeks/No follow-up | No inclusion or exclusion information reported |
| **Koniak-Griffin 2015** | n=223 | To evaluate the effects of a lifestyle behavior intervention delivered by *promotoras* to low-income, overweight immigrant Latinas | RCT | 6-month intervention/3-month follow-up | Self-identify as Latina, Spanish or English-speaker, age 35-64 yrs., and BMI > 25 kg/m^2^  Women were excluded if they had impaired physical mobility, a history of Type 1 DM, or uncontrolled hypertension |
| **Lindberg 2012**  **(*De Por Vida, “For Life”)*** | n=47 | Evaluate the feasibility of developing and implementing a culturally-adapted intervention aimed at the adoption of recommended health behaviors to reduce body weight in MA women | Quasi-experimental (pretest-posttest) pilot study | 12-month intervention/No follow-up | Self-identify as Mexican or MA, Spanish-speaking, 18 yrs. and older, BMI > 30 kg/m^2^ or greater, and PAR-Q (participants with any positive responses were required to obtain medical clearance before enrolling)  Women were excluded if currently pregnant or planning to be or recently gave birth, or moving outside area within 12 months |
| **Marquez 2013**  ***(Comadres Weight Loss Program)*** | n=27 | To evaluate the feasibility and preliminary efficacy of a behavioral weight loss intervention for Latinas that includes a weight loss partner selected from their existing social network | RCT pilot study | 12-week intervention/12-week follow-up | Self-identify as Hispanic or Latino, age 18-65 yrs., BMI between 27-50 kg/m^2^, and report speaking and reading English well  Women were excluded if currently pregnant, > 5% weight loss in past 6 months, or had serious medical condition or psychological disorder |
| **McCurley 2017** | n=61 | To evaluate the feasibility, acceptability, and preliminary effectiveness of a peer-educator-led, culturally appropriate DPP-based lifestyle intervention in Hispanic women at high risk for Type 2 DM | Quasi-experimental (pretest-posttest) pilot study | 6-month intervention/No follow-up | BMI > 25 kg/m^2^  Women were excluded if current diagnosis of diabetes or stroke, inability to engage in light-moderate PA, or planning to move out of the study region |
| **O’Brien 2015** | n=20 | To test the feasibility, acceptability, and preliminary effectiveness of a *promotora*-led DPP in Hispanic women | Quasi-experimental (pretest-posttest) pilot study | 12-month intervention/No follow-up | Age 20 yrs. or greater, BMI > 25 kg/m^2^, Spanish language fluency, and current diagnosis of prediabetes  Women were excluded if currently pregnant or planning to be, current diagnosis of diabetes, chronic conditions affecting ability to engage in PA, had a medical condition affecting weight, taking medications that could affect weight or glucose metabolism |
| **Olvera 2010**  **Olvera 2008**  ***(The BOUNCE Study)*** | n=46 pairs | To assess the efficacy of a family-based exploratory community study to increase physical fitness and activity in low-income Latino mothers and daughters | Cluster RCT | 12-week intervention/No follow-up | No inclusion criteria reported  Women were excluded if current medical condition or dietary restriction |
| **Seguin 2019**  **Perry 2017**  ***(Mujeres Fuertes y Corazones Saludables)*** | n=15 | To collect feasibility and efficacy of a behavioral PA and nutrition intervention | Quasi-experimental (pretest-posttest) pilot study | 12-week intervention/ No follow-up | Age 40-70 yrs., Spanish-speaking, physically inactive, live locally, and PAR-Q (participants with any positive responses were required to obtain medical clearance before enrolling)  Women were excluded for health condition precluding MVPA |
| **Sorkin 2014**  **Sorkin 2013**  ***(Unidas por la Vida)*** | n=89 dyads | To conduct a pilot test of theory-driven, culturally-responsive, behavior lifestyle intervention designed to promote weight loss and improve dietary behavior among high-risk, MA women | RCT pilot study | 16-week intervention/ No follow-up | Age 18 yrs. or older, mother’s residence within 25 miles of daughter’s residence, mothers needed to have current diagnosis of Type 2 DM and daughters needed to have BMI > 25 kg/m^2^ or greater  No exclusion criteria reported |
| **Toobert 2011**  **Toobert 2010**  ***(¡Viva Bien!)*** | n=280 | To document the extent to which the *Viva Bien!* intervention helped Latinas with Type 2 DM make changes in psychosocial factors and multiple lifestyle behaviors | RCT | 24-month intervention/No follow-up | Self-identify as Latina, age 30-75 yrs., current diagnosis of Type 2 DM for at least 6 months, living independently, and have a telephone  Women were excluded if currently on insulin pump, developmentally disabled, or had end-stage renal disease |

**Abbreviations:** PA = physical activity, RCT = randomized controlled trial, PARmedX = Physical Activity Medical Examination, PAR-Q = Physical Activity Readiness Questionnaire, BMI = body mass index, CVD = cardiovascular disease, MA = Mexican American, DM = diabetes mellitus, DPP = Diabetes Prevention Program, MVPA = moderate to vigorous physical activity

**Table S4: Intervention Characteristics of Included Studies**

| **Study** | **Intervention(s) Focus;**  **Sample Size** | **Comparator; Sample Size;**  **Description** | **Intervention Setting;**  **Delivery Modality** | **Intervention Format;**  **Strategies** | **Intervention Goals/**  **Recommendations** | **Culturally Sensitive Intervention Strategies** | **Theoretical**  **Framework** |
| --- | --- | --- | --- | --- | --- | --- | --- |
| **Arredondo 2017**  **(n=436)**  **Arredondo 2015**  **(*Fe en Acciόn, Faith in Action)*** | PA intervention (n=217) | Attention control related to cancer screening (n=219)  6-weekly interactive group discussions (1.5-2 hrs long) led by *promotoras* + 4 optional MI calls over 24 months | **Setting:** Churches, local parks, and community centers  **Delivery Modality:** *Promotora* led intervention | Up to six 1-hr PA classes offered weekly; each PA class was followed by a review of monthly handouts and short discussion on how to apply behavior change strategies; up to three 30-min MI calls  Participants attended as many PA classes as they desired | No specific intervention goals/  recommendations reported | *Promotoras*  Bilingual and bicultural research staff  Training sessions for *promotoras* conducted in Spanish  Church setting  Many classes offered to accommodate various schedules  Images of Latina women in study materials, foods common in Latino diets, music selection, Catholic prayer cards  Pilot tested intervention with Catholic Latina women  Kick-off family night event | Ecological framework for health  behaviors, targeting factors related to the  individual, interpersonal, organizational, and environmental levels |
| **Baldwin 2015**  **(n=48)**  ***(Women’s Path to Prevention (WPP))*** | Multicomponent diet + PA intervention | Not applicable | **Setting:** NR (described as urban Hispanic neighborhood)  **Delivery Modality:** Nursing students led intervention | 12 weekly nutrition and wellness education and coping skills training classes; included walking assignments, individual health coaching, and MI groups  Participants given glycemic index information, low-carb cookbook, PA activity plan, journal, and walking maps  Focus on goal setting and plans of action | Participants given pedometers and were encouraged to record time and steps/week in daily logs  Participants were expected to attend program’s low-intensity classes for 2 hrs/week and expected to walk 3 hrs/week | Bilingual students  Translated materials | No specific theory reported (adapted from National Diabetes Education Program) |
| **Berry 2011**  **(n=56)** | Diet + PA + coping skills training intervention + *Color Me Healthy* intervention for children | Wait-list control | **Setting:** Local church and community center in neighboring town  **Delivery Modality**: Community health educator and *promotora* led intervention | Weekly 60-minute nutrition and exercise education and coping skills training classes followed by a 45-minute exercise class (12 weeks), once per month classes for 3 months, then 3 months on their own  Focus on coping skills, social problem solving, conflict resolution, and cognitive restructuring | Mothers received a pedometer as part of the intervention and were asked to record daily steps  Encouraged to walk 30–60 min/day on most days of the week  Mothers were asked to increase their steps by 500/day for the first week and then continue to increase their steps by 500/week until they were averaging 10,000 steps/day  Nutrition education focused on healthy, low-cost food choices, portion control, and lowering fat and calories for mothers  Mothers asked to share a nutrition or exercise goal to work on for the coming week | *Promotoras*  Bilingual staff  Intervention was refined, adapted, and then translated at a low literacy level  Intervention delivered in Spanish  Classes included examples like traditional meals made healthier and at-home exercises due to weather and safety concerns  Zumba during PA classes  Small walking groups with local partners | SCT |
| **Cousins 1992**  **(n=168)** | Diet + PA intervention  IG (sample size NR) vs. FG (sample size NR) | Manual only group (sample size NR)  Bilingual manual (“Cuidando el Corazon”), consisting of a low-fat eating  plan, nutrition information, recipes, an exercise  plan, and behavior modification strategies, based  on a previously developed program and modified  to reflect the cultural values of the population | **Setting:** NR  **Delivery Modality**: Bilingual registered dietitians led both the individual and family group intervention | IG: “Cuidando el Corazon” manual + 24 weekly classes (individualized nutrition, feedback on subject’s food records, behavior change techniques, included group exercise, food tastings, cooking demos, and low-fat food videos) + 6 monthly maintenance classes  FG:  IG intervention with a modified version of the “Cuidando el Corazon” manual that  included information on partner support and parenting  skills to encourage family changes in eating and exercise behaviors  Focus on goal setting, portion control, problem solving and relapse prevention | Calories were limited to 1,200/day for women  Fat intake to comprise 30% of total kcals, saturated fat < 10% of total kcals, carbohydrates about 50% of total kcals, protein about 20% of total kcals, cholesterol limited to 300 mg/d, sodium limited to 1,200 mg/d | Bilingual registered dietitian  Inclusion of family members  Translated materials  Bilingual manual modified to reflect cultural values of the population - evaluated by bilingual health educators and members of the MA community  Cookbook prepared with recipes for fat-modified traditional MA recipes | No specific theory reported |
| **Faucher 2010**  **(n=24)** | Diet intervention (n=13) | Standard care counseling (n=11)  One-time counseling on weight loss included a physical exam and education on portion control and exercise by a *promotora* and a primary care provider (primary researcher) | **Setting**: NR  **Delivery Modality:** Primary researcher, a research assistant, and *promotora* led intervention | Four 2-hr group meetings at weeks 1, 3, 7, and 13 focused on portion control including meal sampling, recipe sharing, use of portion control aids measuring  aids, and portion control plates | No specific intervention goals/  recommendations reported | *Promotoras*  Visits translated from English to Spanish  Translated materials    Curriculum designed in a culturally and economically-sensitive manner  Importance of health for the whole family  Foods prepared for classes were MA food, low cost, quick to prepare | No specific theory reported |
| **Harralson 2007**  **(n=225)**  ***(Un Corazon Saludable: A Healthy Heart)*** | PA intervention | Not applicable | **Setting:** Latino-owned gym located in mainly Puerto Rican neighborhood  **Delivery Modality**: NR | 1-hr exercise classes 3x/week + 30-min education modules 1x/week  Exercise class content was based on recommendations from the ACSM  Educational modules based on a materials by AHA and NHLBI | No specific intervention goals/  recommendations reported | Translated materials  Modification of favorite Latino recipes  Salsa dancing | No specific theory reported |
| **Koniak-Griffin 2015**  **(n=223)** | Diet + PA intervention (n=111) | Attention Control related to safety/disaster training (n=112)  6-month educational program consisting of 8 group classes focused on safety/disaster education conducted by separate team of *promotoras* followed by 8 contacts of Individual Teaching Coaching to discuss class material | **Setting**: Community settings like school classrooms and home visits  **Delivery Modality:** *Promotora* led intervention | 8 weekly 2-hr group education classes for the first 2 months followed by 4 months of SCT and Coaching  Contacts (4 home visits plus 4 phone calls delivered over 4 months)  The first 8 classes were based upon *Your Heart, Your Life or Su Corazon, Su Vida*, a culturally-adapted education program developed for Latinos by the NHLBI (2008)  Focus on goal setting, portion control, managing emotional eating, and self-monitoring | Participants given pedometers and exercise DVD and were encouraged to record steps per week of PA in daily logs  Use of food diaries was encouraged  Participants established personal goals for lifestyle changes. Four key messages were emphasized: (1) healthy food choices, (2) portion control, (3) managing emotional eating, and (4) increasing physical activity, with the goal of walking 10,000 steps/day | *Promotoras*  Bilingual RAs and  bicultural staff  Lifestyle Behavior Intervention implemented in Spanish  Culturally-tailored education materials (language, healthy traditional Latino meals, etc.)  Flexible scheduling  Use of a CBPR approach including CAB involvement | No specific theory reported |
| **Lindberg 2012**  **(n=47)**  **(*De Por Vida, “For Life”)*** | Diet + PA intervention | Not applicable | **Setting:** Local health care facility  **Delivery Modality**: Intervention delivered by 2 Spanish-speaking Mexican women who were clinicians | Phase 1 consisted of 6 months of weekly 90-min group sessions followed by 6 months of monthly 90-min group sessions (Phase 2)  Focus on goal-setting, developing specific behavior-change plans, monitoring progress, and problem-solving | Participants given CalorieKing^TM^ guide and list of 50 commonly consumed foods with calorie content in Spanish  Participants encouraged to keep food diaries – taught to tally number of servings of different food groups  Dietary and PA recommendations in the program were consistent with the 2005 Dietary Guidelines for Americans | Women only participants  Bilingual and bicultural female interventionists  Enrollment visit conducted in a group setting  Minimal written materials  Small and large-group activities  PA for the home  Focus on Mexican traditions  Salsa dancing  Food measurement  Food journaling for limited literacy  Traditional beliefs regarding health and food  Topics central to immigration experience | Intervention modeled after PREMIER Trial and Weight Loss Maintenance Trials  SCT (Self-Management) |
| **Marquez 2013**  **(n=27)**  ***(Comadres Weight Loss Program)*** | Diet + PA intervention  PLG (n=13) | Both study conditions received the same culturally-adapted version of DPP, however ILG participants did not have a weight loss partner (n=14) | **Setting:** Research center  **Delivery Modality**: Intervention co-facilitated by registered dietitian and doctoral-level interventionist | 12 1-hr weekly, group-based sessions  Focus on improving partner communication and collaboration, goal setting, planning ahead, problem solving, stimulus control. cognitive restructuring,  relapse prevention, and self-monitoring | Participants given goal of 1,200 or 1,500 kcals/day for < 91 kg or > 91 kg respectively; recommended  <30% calories from fat  Participants given goal to increase PA gradually to >30 min/day of moderate intensity 5d/week, goal of 10,000 steps/day  Participants asked to record food (energy and fat) intake, PA, pedometer steps, and body weight in diaries  Participants in PLG encouraged to engage in PA + diet-related activities together | Women only participants  All female-staff  Bilingual/bicultural Latina interventionist  Promoted walking and dancing  Inclusion of close friend or “comadre”  Encouraged consumption of traditional meals and modified cooking methods – participants received a booklet with fat kcal content of ethnic foods | No specific theory reported (modeled after DPP) |
| **McCurley 2017**  **(n=61)** | Diet + PA intervention | Not applicable | **Setting:** Community settings  **Delivery Modality**: *Promotora* led intervention | 12 weekly, 2-hr group-based, educational lifestyle classes followed by  maintenance sessions offered Month 3 – Month 6 to review intervention materials and increase social support  Focus on self-monitoring, goal setting, self-efficacy,  social support, social modeling of healthy behaviors, stress and negative emotion coping skills,  autonomous decision making, and enjoyment and “fun as motivators | 5% weight loss goal | *Promotoras*  Bilingual staff  Translated materials    Latino cultural elements (foods, beliefs, etc.) educational content  Promoted walking and dancing  Didactic content tailored to participant language, culture, SES, using local *promotora* expertise and feedback throughout the adaption process | SCT  Self-Determination Theory  Intervention modeled after DPP |
| **O’Brien 2015**  **(n=20)** | Diet + PA intervention | Not applicable | **Setting:** Community-based organization serving Latinos  **Delivery Modality**: *Promotora* led intervention | Group-based lifestyle intervention delivered over 24 sessions; first 14 weekly sessions then remaining 10 delivered bi-weekly and then monthly  Focus on self-monitoring and self-management | Participants were given a weight loss goal of 7% initial body weight, a pedometer, and a goal of 150 min/week moderate PA  Participants asked to record fat and kcal intake, daily steps, and weight | *Promotoras*  Sessions conducted in Spanish  Translated materials  Latino family context for healthy behavior change  Culturally-appropriate tools for dietary self-monitoring | No specific theory reported (modeled after DPP) |
| **Olvera 2010**  **(n=46 pairs)**  **Olvera 2008**  ***(The BOUNCE Study)*** | Diet + PA intervention  (n=26 pairs) | 12 weekly 1.5 hr. meetings with an instructor which included a review of educational handouts and 45 min. of light intensity aerobic or sport PA sessions (n=20 pairs) | **Setting:** Community settings like parks and schools  **Delivery Modality:** A child psychologist and licensed counselor, registered dietitian/nutrition educators, and trained fitness specialists led intervention | Two 45-minute sessions of group nutrition education/week, one 45-minute group behavioral counseling session per week, and three 45-minute group exercise sessions per week  (6 classes/week for 12 weeks)  Focus on learning a new skill, self-efficacy, expecting positive outcomes, self-control by goal setting, self-monitoring, problem solving, stimulus control, management plans that included intrinsic and extrinsic rewards, and role modeling | Behavioral component had 3 goals: 1) create awareness of positive physical attributes to enhance self-acceptance, 2) address perceptions and distorted thoughts about food, body weight, and exercise, and 3) teach effective communication, problem solving techniques, and coping strategies to handle new challenging situations  Nutrition education had 3 goals: 1) reduce intake of sugary beverages while increasing water consumption; 2) reduced intake of saturated fat; and 3) develop healthy eating strategies when dining away from home | Latino bilingual instructors  Lessons taught in Spanish or English based on preference  Translated materials  Latino mother-daughter dyads  Focus on dance  Nutrition and cooking lessons tailored to Latino population (food, music)  Used acculturation level to tailor program planning  Formative assessment included piloting materials with community members and revising | SCT |
| **Seguin 2019**  **(n=15)**  **Perry 2017**  ***(Mujeres Fuertes y Corazones Saludables)*** | Diet + PA intervention | Not applicable | **Setting:** Rural community organization serving Latinas  **Delivery Modality:** Bilingual community health educators | Two 60-min classes held 2x/week for 12 weeks; included 30 min of PA and 30 min of nutrition education  Nutrition classes focused on following a heart healthy diet, self-monitoring and SMART goals, group cohesion activities, and skill building  Focus on self-efficacy and social support | Participants were given goal of increasing PA up to 150 min/week  Participants encouraged to keep exercise and food logs  Behavioral goals included the following: 1) prepare healthy meals, 2) reduce refined carbohydrate intake, 3) reduce fat intake, and 4) consume five fruits & vegetables per day | Bilingual class leader  Translated materials  Culturally-tailored foods and recipes  Tailoring of materials to increase cultural relevancy including emphasis on family and social and economic barriers  Latin dancing  Family celebration at the end of intervention  Use of CBPR approach with CAB | SCT |
| **Sorkin 2014**  **(n=89 dyads)**  **Sorkin 2013**  ***(Unidas por la Vida)*** | Diet + PA intervention (n=53 dyads) | Comparison group participants (n=36 dyads)  were mailed educational materials from the National Diabetes Education Program | **Setting:**  Group meetings (setting not specified), home visits  **Delivery Modality**: Lifestyle community coach led intervention | Mother-daughter dyads attended 4 group meetings which consisted of recipe demo and 20 min exercise, 8 home visits with 4 booster telephone calls between home visits  Focus on self-monitoring, goal setting, problem solving, and relapse prevention; techniques adapted to encourage dyadic collaboration | Participants given personal weight loss goal to be achieved via reduction in caloric intake (1200-1800 kcals/day) and moderate PA (> 150 min/week)  Participants were encouraged to record diet and PA daily | Translated materials  Mother-daughter dyads  Recipe demos of modified Hispanic dishes  Zumba and salsa dancing for PA | No specific theory reported (modeled after DPP) |
| **Toobert 2011**  **(n=280)**  **Toobert 2010**  ***(¡Viva Bien!)*** | Diet + PA intervention (n=142) | Standard care (n=138)  In compliance with ADA + a choice of 1 free Kaiser-Permanente class covering the areas targeted in the intervention | **Setting:** Community settings  **Delivery Modality:** Facilitators led intervention | Months 1-6 = weekly 4-hr meetings included  1 hr. of each Mediterranean diet potluck, PA, stress management, and social support groups  Months 7-12 = (above) semi-monthly  Months 12-18 = (above) monthly  Months 18-24 = (above) bi- monthly  Focus on problem-solving, self-efficacy, and social support | Goals of the intervention were to encourage participants to  a) Follow Mediterranean diet adapted for Latinas  b) Practice stress-management techniques daily  c) Engage in 30 min daily PA  d) Stop smoking  e) Participate in problem-solving-based support groups | Culturally-adapted from an established lifestyle change program  Sessions held in Spanish and/or English  Colorful translated intervention pamphlets included pictures of Latinas  Flexible scheduling  Added a retreat at the start of the program  Family night to celebrate achievements  Latina dietitian  Latin dancing  Take-home intervention DVDs and CDs offered | No specific theory reported |

**Abbreviations:** PA = physical activity, MI = motivational interviewing, NR = not reported, SCT = Social Cognitive Theory, IG = Individual Group, FG = Family Group, MA = Mexican American, ACSM = American College of Sports Medicine, AHA = American Heart Association, NHLBI = National Heart, Lung, and Blood Institute, RA = research assistant, CBPR = community-based participatory research, CAB = community advisory board, PLG = Partner Lifestyle Group, ILG = Individual Lifestyle Group, DPP = Diabetes Prevention Program, SES = socioeconomic status, ADA = American Diabetes Association

**Table S5: Outcomes of Included Studies**

| **Study; Sample Size** | **Study Participants (age (SD)), Hispanic/Latino subgroup, measures of acculturation)** | **Outcomes; Data Collection Time Points** | **Weight change (kg or BMI)** | **Other Outcomes; Additional Significant Findings** | **Retention and Adherence Strategies** | **Retention Figures;**  **Adherence/Attendance Figures** |
| --- | --- | --- | --- | --- | --- | --- |
| **Arredondo 2017**  **(n=436)**  **Arredondo 2015**  **(*Fe en Acciόn, Faith in Action)*** | Differences between groups n.s. (below represents total sample):  Mean age: 44.4 yrs. (9.6)  90.8% born in Mexico  Average time in U.S: 21 yrs. (10.3) | **Primary:** Accelerometer-assessed PA and self-reported PA  **Secondary:** BMI, WC, 3-min step test, behavioral strategies for PA  **Time points:** Baseline, 12 months (reported here), 24 months | Weight change in kg NR  **Mean BMI change from Baseline at 12 months**:  Intervention: -0.1 kg/m^2^ (1.9); (Sig. NR)  Control: +0.4 kg/m^2^ (1.5); (Sig. NR)  Mean difference in BMI between conditions at 12 months: adjusted mean = -0.43 kg/m^2^; (P=.04) | Sig. increases in accelerometer and self-reported PA, proportion of participants meeting 2008 PAG, and behavioral strategies for engaging in PA compared to control at 12 months  Greater class attendance associated with sig. increases in self-reported MVPA, adherence to 2008 PAG, and WC  Greater likelihood of meeting 2008 PAG at 12 months with every additional MI call completed | Kick-off family night included dinner, presentation, and introduction to *promotoras*  *Promotoras* called absent participants and encouraged them to attend future classes  Monthly raffles  Financial incentives for each evaluation | **Retention**:  At 12 months for total sample = 87% (n=380)  Differences between conditions n.s.  **Attendance:**  The average number of classes attended per month ranged from 0 to 13, with about 40% having attended 0 classes |
| **Baldwin 2015**  **(n=48)**  ***(Women’s Path to Prevention (WPP))*** | Mean age: 58 yrs. (32)  No measures of acculturation reported | **Primary:** HbA1c, BMI, WHR, BP  **Secondary:** Wellness health behaviors and diabetes and CVD risk factors  **Time points:** Baseline, 12 weeks | Mean weight change in kg NR  **Mean BMI change from Baseline:** -3.22 kg/m^2^; (p<.05); SD NR | Sig. improvements in HbA1c, health behavior total risk score, and PA behaviors | None reported | **Retention**:  25% drop out rate  Attendance figures NR |
| **Berry 2011**  **(n=56)** | Differences between groups n.s. (below represents total sample):  Mean age: 29.7 yrs. (5.3)  100% Foreign-born (Mexico)  100% Spanish- speaking | **Primary**: BMI of mother  **Other:** WC, skinfolds, fasting glucose, insulin, lipids,  frequency of health promoting behaviors, self-efficacy for eating and exercise  **Time points:** Baseline, 9 months | Mean weight change in kg NR  **Mean BMI change from Baseline:**  Intervention: -3.0 kg/m²  Control: +1.0 kg/m²  Weight changes above calculated from Table 1; Sig. and SDs NR  Differences in weight change between conditions n.s. | Mothers experienced sig. improvements in subscapular skinfolds, fasting blood glucose, eating self-efficacy, exercise self-efficacy, and nutrition, and exercise knowledge | Transportation provided for collection visits  A healthy snack was served at each class  Childcare was always available  Financial incentives for each evaluation | Retention and attendance figures NR |
| **Cousins 1992**  **(n=168)** | Differences between groups n.s. (below represents total sample):  Mean age: 33.4 yrs. (6.4)  100% MA  ARSMA identified sample as “truly bicultural” | **Primary:** weight and BMI change  **Secondary:** None reported  **Time points:** Baseline, 3 months, 6 months, 12 months | **IG weight change from Baseline:**  3 months: -2.6 kg  6 months: -3.3 kg  12 months: -2.1 kg  **FG weight change from Baseline**:  3 months: -3.0 kg  6 months: -4.5 kg  12 months: -3.8 kg  **Control change from Baseline:**  3 months: -0.9 kg  6 months: -0.2 kg  12 months: -0.7 kg  Weight changes above calculated from Table 2; Sig. and SDs NR  Differences in weight loss between intervention groups n.s.  Both were sig. compared to control | Not applicable | None reported | **Retention**:  At 12 months for total sample = 51.2% (n=86); Did not report per group  Attendance figures NR (although husbands were encouraged to attend FG classes, fewer than 50% attended any) |
| **Faucher 2010**  **(n=24)** | Differences between groups n.s (below represents total sample):  Mean age: 34.9 yrs. (4.8)  94.7% MA, 5.3% Honduran  94.7% Spanish primary language  Average time in U.S. (months): 108.6 (50.9) | **Primary:** Weight change  **Secondary:** None reported  **Time points**: Baseline and 20 weeks | **Mean weight change from Baseline**:  Intervention: -6.5 lbs (7.5); n.s.  Control: -2.8 lbs (10.2); n.s.  Differences in weight loss between conditions n.s. | Not applicable | Incentives for classes, retention and  follow-up attendance  Both groups received  a refrigerator magnet and postcards with a motivational message about  weight loss  Raffle at final meeting | **Retention:**  Intervention = 63.6% (n=7)  Control = 55.5% (n=5)  Attendance figures NR |
| **Harralson 2007**  **(n=225)**  ***(Un Corazon Saludable: A Healthy Heart)*** | Mean age: 44.1 yrs. (SD NR)  74% born in Puerto Rico, 17% U.S., and 9% other Latin America | **Primary:** BMI, WHR, BP  **Other:** Perceived support and stress levels, depression scores, health knowledge, and self-rated health  **Time points:**  1^st^ year of the intervention = Baseline and 12 weeks  2^nd^ year of the intervention = Baseline and 16 weeks | **Mean weight change from Baseline**:  -2 lbs; (Sig. and SD NR)  A sig. decrease was reported for BMI (magnitude of change NR) | Sig. decreases in abdominal obesity, WHR, symptoms of depression  Sig. improvement in self-rated health | Financial incentives for each evaluation  Raffle during classes  Childcare and tokens for transportation provided | **Retention**: 52% (n=118)  Completers compared to non-completers were sig. more likely to be older, foreign-born and sig. less likely to be a caregiver, receiving public assistance, have higher levels of stress, and have lower depression scores  Attendance figures NR |
| **Koniak-Griffin 2015**  **(n=223)** | Intervention:  Mean age: 43.3 yrs. (7.4)  Control: Mean age: 45.9 yrs. (8.2)  Groups did not differ in the below baseline measures:  83.9% born in Mexico, 1.8% born in U.S. but raised in Mexico, 14.3% Dominican, Central or South American  54.3% Spanish only language  Mean years in U.S.: 18.6 yrs. (8.3)  5-item scale (Balcazar) showed low acculturation despite lengthy U.S. residence | Primary vs. secondary outcomes not distinguished  Outcomes included BMI, weight, WC, BP, blood lipids and glucose, dietary habits, objective measure of PA, and knowledge of heart disease  **Time points:** Baseline, 6 months, 9 months | **Intervention weight change from Baseline**:  6 months:  -1.46 lbs; n.s.  9 months:  -2.25 lbs; n.s.  **Control weight change from Baseline:**  6 months:  -2.78 lbs; n.s.  9 months:  +0.15 lbs; n.s.  Weight changes above estimated from Table 2; Sig. and SDs NR  Changes from Baseline (within group and between groups) n.s. | Sig. decrease in WC from Baseline at 9 months  Sig. improvements in dietary habits at 6 months and maintained at 9 months (both from Baseline and compared to control)  Sig. increase in daily step count from Baseline to 9 months compared to control however difference in daily minutes of moderate PA from Baseline to 9 months between groups n.s.  Sig. differences from Baseline were found for BMI, weight, and WC for those receiving a higher intervention dose compared to those receiving a low intervention dose | Case management approach to build rapport  Telephone reminders for classes and evaluations  Financial incentives for each evaluation  Small gifts for class attendance  Providing participants a record of their health (weight, BP, lipids)  Flexible scheduling  Frequent contacts of *promotoras* with participants  Child care and bus tokens provided | **Retention:**  At 6- and 9-months for total sample = 86.5% (n=193) and 87% (n=194), respectively  Differences between conditions n.s.  **Attendance:**  8 weekly Classes:  42 (37.8%) attended all classes,  91 (82%) attended at least half  Individual Teaching Classes: 86 (77.5%) received targeted number of home visits (4)  31 (27.9%) received all component of the intervention |
| **Lindberg 2012**  **(n=47)**  **(*De Por Vida, “For Life”)*** | Mean age**:** 38 yrs. (11.7)  100% MA  89% born in Mexican, 11% born in U.S.  100% Preferred Spanish language | Primary vs. secondary outcomes not distinguished  Outcomes included weight loss and dietary intake  **Time points:** Baseline, 6 months, 12 months | **Mean weight change from Baseline**:  6 months: -5.3 kg (5.3); (p<.0001)  12 months: -7.2 kg (6.8); (p<.0001)  74% of all participants lost > 5% of initial weight | Phase 1 (Months 1-6): Sig. decrease in BMI, total kcals, % total calories from fat, and refined sugar  Phase 1: Sig. increases in % total calories from protein and % total calories from carbohydrates, number of daily servings of vegetables | None reported  Study made to mirror “real world” setting | **Retention:**  Months 1-6: 66% (n=31)  Months 7-12: 55% (n=26)  **Attendance:**  Months 1-6: 62%  Months 7-12: 58% |
| **Marquez 2013**  **(n=27)**  ***(Comadres Weight Loss Program)*** | Differences between groups n.s. (age represents total sample):  Mean age: 43.0 yrs. (10.2)  Acculturation  measures did not differ between groups but were reported separately:  ILG:  35.7% Dominican, 14.3% Puerto Rican, 7.1% Colombian, 42.9% other  21.4% More Spanish than English  64.3% First generation, 28.6% Second generation  Mean years in U.S.: 25.2 (15.3)  PLG:  30.8% Dominican, 30.8% Puerto Rican, 23.1% Colombian, 15.3% other  15.4% More Spanish than English  69.2% First generation, 30.8% Second generation  Mean years in U.S.: 32.5 (6.9)  For total sample:  66% Foreign-born (mostly Caribbean) | **Primary**: Treatment adherence, study retention, and weight change  **Secondary**:  Changes in psychosocial variables and self-reported PA  **Time points:** Baseline, 12 weeks, 24 weeks | **Intervention weight change from Baseline:**  12 weeks:  -4.3 kg (4.4); (p<0.01)  24 weeks: -4.7 kg (5.0); (p<0.01)  **Control weight change from Baseline**:  12 weeks:  -4.7 kg (4.2); (p<0.01)  24 weeks:  -5.0 kg (6.4); (p<0.01)  Differences in weight loss between groups n.s.  Almost 50% achieved > 5% weight loss | Both groups experienced sig. increases in weight loss efficacy, exercise self-efficacy, and family social support for exercise habits at 12 weeks but only weight loss self-efficacy was maintained at 24 weeks  Across groups, positive relationship between weight loss at 12 weeks with number of sessions attended, number of diaries submitted, kcals expended, weight loss self-efficacy and friend social support for eating habits | Female weight-loss partner  Financial incentives for each evaluation | **Retention:**  At 12 weeks for total sample: 96% (n=26)  At 24 weeks for total sample: 100% (n=26, 1 became pregnant)  **Attendance for total sample:**  Participants attended 70% of 12 sessions  **Adherence for total sample**:  Participants submitted 68% of self-monitoring diaries  Differences in attendance and adherence between groups n.s. |
| **McCurley 2017**  **(n=61)** | Mean age: 47.8 yrs. (10.8)  91.2% born in Mexico, 6.8% born in U.S., 2.1% other | **Primary:** Weight change and glucose control  **Secondary:**  Self-reported dietary behaviors, self-reported PA, barriers to health behavior change, stress, depression symptoms  **Time points:** Baseline, 3 months, 6 months | **Mean weight change from Baseline:**  3 months: -7 lbs; n.s.  6 months:  -6.6 lbs; n.s.  Weight changes above estimated from Table 2; SDs NR  21.6% of women achieved 5% weight loss goal | Sig. improvements in dietary behaviors, perceived stress, and depression symptoms  Improvements for any clinical indicators n.s. | Shorter intervention time-frame (12 sessions)  Child care provided (Dulce Mothers) | **Retention:**  6-months: 95% (n=58)  **Attendance:**  Participants attended an average of 8.02 (3.33) of the 12 classes  72% attended at least 6 classes  11.5% attended all 12 classes |
| **O’Brien 2015**  **(n=20)** | Mean age: 44.5 yrs. (13.0)  100% Foreign- born  40% born in Mexico, 40% born in Caribbean, 15% born in Central America, 5% born in Other  100% Spanish language dominant  Average years in U.S.: 18.9 yrs. (14.0) | **Primary:** Weight change  **Secondary**: WC, BP, HbA1c, fasting glucose, insulin, lipids  **Exploratory**: Health literacy, health-related quality of life, social support, stress, and depression and anxiety as mediators or moderators of weight loss  **Feasibility:** Attendance, attrition, acceptability  **Time points:**  Baseline and 12 months | **Mean weight change from Baseline:**  -10.8 lbs (95% CI: -5.6, -16.0); (p<.001)  42% achieved 7% weight loss goal; 58% achieved > 5% weight loss | Sig. decreases in WC, diastolic BP, LDL, fasting insulin  As exploratory, depression symptoms sig. decreased  Weight change sig. correlated with perceived stress and anxiety  Session attendance was sig. associated with weight loss | None reported | **Retention:**  12 months: 95% (n=19)  **Attendance:**  Participants attended average of 71% of sessions  90% of completers attended at least 12 sessions  15% of completers attended all 24 sessions |
| **Olvera 2010**  **(n=46 pairs)**  **Olvera 2008**  ***(The BOUNCE Study)*** | Intervention:  Mean age of mothers:  33.3 yrs. (4.6)  Mean age of daughters: 9.9 yrs. (1.1)  Control:  Mean age of mothers: 38.2 yrs. (10.6)  Mean age of daughters: 10.4 yrs. (1.1)  Groups did not differ in the below baseline measures:  82% of daughters born in U.S.  100% of mothers born in Mexico or Central America  62% of mothers preferred Spanish language  Maternal preferred language used as acculturation measure  For daughters, Short Acculturation Scale for Latino Youth showed 85% of girls reported low acculturation | **Primary:** physical Fitness, daily counts per min. of MVPA  **Secondary:** BMI, dietary fat intake, intake of sugary beverages, fruit and vegetable consumption  **Time points:** Baseline, 12 weeks | Weight change in kg NR  Decreases in BMI n.s. (magnitude of change NR) | Changes in physical fitness or self-reported PA levels in mothers n.s.  Sig. higher levels of physical fitness in daughters compared to control  Changes in av. daily counts per min of MVPA for daughters compared to control n.s. | Participants received binders with handouts and take home recipes, BOUNCE t-shirts, BOUNCE water bottle  Small gifts for class attendance  If participants missed a session, they were called and received missing info by mail | **Retention:**  At 12-weeks for total sample: 76% (n=35 pairs)  Differences in retention between conditions n.s.  **Attendance:**  Intervention:  Daughters: 61% of sessions  Mothers: 56% of sessions  Control:  Daughters: 64% of sessions  Mothers: 50% of sessions |
| **Seguin 2019**  **(n=15)**  **Perry 2017**  ***(Mujeres Fuertes y Corazones Saludables)*** | Mean age: 52.2 (SD NR)  Short Acculturation Scale for Hispanics showed 71.4% reported low acculturation | Primary vs. secondary outcomes not distinguished  Outcomes included weight, BMI, WC, cardiorespiratory fitness, self-reported PA, dietary behavior, self-efficacy for diet and PA  **Time points:** Baseline, 12 weeks | **Mean weight change from Baseline:**  -1.5 kg (-2.5, -0.5); (p=0.009) | Sig. decreases in WC and sugary drinks  Sig. increase in fruits and veggies intake  Sig. improvements in 6-min walk test and PA self-efficacy | Close contact maintained with all participants by class leaders  Reminder calls before the day of visit  Class leaders called participants who missed class to create a plan to overcome barriers | **Retention:**  12 weeks: 73% (n=11)  **Attendance:**  Participants attended an average of 62% of classes |
| **Sorkin 2014**  **(n=89 dyads)**  **Sorkin 2013**  ***(Unidas por la Vida)*** | Differences between groups n.s. (below represents total sample):  Mean age of mothers: 52.7 yrs. (6.9)  Mean age of daughters: 27.8 yrs. (7.4)  100% MA  95% of mothers and 63% of daughters Foreign-born  73% of mothers and 11% of daughters Spanish language only | **Primary:** Feasibility outcomes (attendance to sessions and completion of home visits, acceptability)  **Secondary:** weight loss, changes in psychosocial measures  **Time points**: Baseline, 16 weeks | **Mean weight change from Baseline:**  Intervention:  -3.5 lbs (mothers)  -4.6 lbs (adult daughters)  Control:  +1.3 lbs (mothers)  -1.6 lbs (adult daughters)  Weight changes above calculated from Table 1; Sig. and SDs NR  Intervention group experienced sig. decreases in weight compared to control (p<0.003) | Sig. decreases in glycemic load and saturated fat intake compared to control  Sig. increases in health-related social support and persuasion compared to control | None reported | **Retention:**  At 16 weeks for total sample:  96.1% (n=171)  Differences between conditions n.s.  **Attendance**:  Intervention: 73% of sessions  Participants were more likely to complete home visit (average 7/8) vs. group visits (average 2.2/4) and booster phone calls (2.5/4) |
| **Toobert 2011**  **(n=280)**  **Toobert 2010**  ***(¡Viva Bien!)*** | Intervention:  Mean age: 55.6 yrs. (9.7)  Control: Mean age: 58.7 yrs. (10.3)  Groups did not differ in the below baseline measures:  15.8% Foreign- born  16% Spanish preferred Speaker  79.6% born in USA  ARSMA-II and generation status showed 40.8% reported mostly Anglo-oriented | Primary vs. secondary outcomes not distinguished  Outcomes included BMI, problem-solving ability, self-efficacy, social support, % total calories from saturated fat, stress-management practice scores, self-reported PA, perceived social-environment support for disease-management, HbA1c  **Time points:** 6 months, 12 months, 24 months | Weight change in kg NR  **Intervention BMI change from Baseline:**  6 months: -0.8 kg/m^2^ (SE NR); (p<0.05)  12 months: -1.1 kg/m^2^ (SE NR); (p<0.05)  24 months: -0.4 kg/m^2^ (SE NR); (p<0.05)  **Control BMI change from Baseline:**  6 months: -0.9 kg/m^2^ (SE NR); (p<0.05)  12 months: -0.4 kg/m^2^ (SE NR); (p<0.05)  24 months: -0.5 kg/m^2^ (SE NR); (p<0.05)  Intervention group experienced sig. decreases in BMI compared to control (p<0.05) | Sig. improvements in problem solving,  self-efficacy, and perceived support in intervention compared to control at 6 months– these were maintained at 12 and 24 months  Sig. improvements in self-management and PA compared to control at 6 months were not maintained (control PA increased)  Sig. reductions in % total kcal from sat fat and engagement in social-environment support activities compared to control across 24 months  No maintenance of lifestyle or biologic outcomes | Family member involvement  Flexible assessment times  Follow-up reminders  Friendly competitions  Social connection with staff and peers encouraged  Phone calls and cards sent to missing participants  Taxi service to minimize transportation barriers | **Retention of Intervention participants**:  6 months: 77.5% (n=217)  12 months: 70% (n=197)  24-months: 61.4% (n=172)  Differences between conditions n.s.  **Attendance of Intervention participants:**  0-6 months: 65%  6-12 months: 48%  12-24 months: 46%  Completers were more likely to be older and have higher health literacy (did not differ in BMI, income, education, language preference, acculturation) |

**Abbreviations:** SD = standard deviation, U.S. = United States, PA = physical activity, BMI = body mass index, WC = waist circumference, PAG = physical activity guidelines, MI = motivational interviewing, NR = not reported, MVPA = moderate to vigorous physical activity, HbA1c = hemoglobin A1c, WHR = waist-to-hip ratio, BP = blood pressure, CVD = cardiovascular disease, MA = Mexican American, ARSMA = Acculturation Rating Scale for Mexican Americans, IG = Individual Group, FG = Family Group, ILG = Individual Lifestyle Group, PLG = Partner Lifestyle Group, LDL = low-density lipoprotein, ARSMA-II = Acculturation Rating Scale for Mexican Americans – II, SE = standard error

**Table S6: Risk of Bias and Quality Assessment of Included Studies**

| **Study** | **Selection Bias** | **Study Design** | **Confounders** | **Blinding** | **Data Collection Methods** | **Withdrawals and Dropouts** | **Overall Quality Score** |
| --- | --- | --- | --- | --- | --- | --- | --- |
| **Arredondo 2017** | Weak | Strong | Strong | Moderate | Strong | Strong | Moderate |
| **Baldwin 2015** | Weak | Moderate | Weak | Moderate | Strong | Moderate | Weak |
| **Berry**  **2011** | Weak | Strong | Strong | Moderate | Strong | Weak | Weak |
| **Cousins 1992** | Weak | Strong | Strong | Moderate | Strong | Weak | Weak |
| **Faucher 2010** | Weak | Strong | Strong | Moderate | Weak | Weak | Weak |
| **Harralson 2007** | Weak | Moderate | Weak | Moderate | Weak | Weak | Weak |
| **Koniak-Griffin 2015** | Weak | Strong | Strong | Moderate | Strong | Strong | Moderate |
| **Lindberg 2012** | Weak | Moderate | Weak | Moderate | Strong | Weak | Weak |
| **Marquez 2013** | Weak | Strong | Strong | Moderate | Weak | Strong | Weak |
| **McCurley 2017** | Weak | Moderate | Strong | Moderate | Strong | Strong | Moderate |
| **O’Brien 2015** | Weak | Moderate | Weak | Moderate | Strong | Strong | Weak |
| **Olvera 2010** | Weak | Strong | Strong | Moderate | Strong | Moderate | Moderate |
| **Seguin 2019** | Weak | Moderate | Weak | Moderate | Strong | Moderate | Weak |
| **Sorkin 2014** | Weak | Strong | Strong | Moderate | Strong | Strong | Moderate |
| **Toobert 2011** | Weak | Strong | Weak | Moderate | Strong | Moderate | Weak |
